# RNA sequencing revealed novel actors of the acquisition of drug resistance in *Candida albicans*

**DOI:** 10.1186/1471-2164-13-396

**Published:** 2012-08-16

**Authors:** Sanjiveeni Dhamgaye, Maria Bernard, Gaelle Lelandais, Odile Sismeiro, Sophie Lemoine, Jean-Yves Coppée, Stéphane Le Crom, Rajendra Prasad, Frédéric Devaux

**Affiliations:** 1 Membrane Biology Laboratory, School of Life Sciences, Jawaharlal Nehru University, New Delhi 110067, India; 2 UPMC, UMR7238, Génomique des Microorganismes, 15 rue de l'École de Médecine, 75006, Paris, France; 3 Ecole Normale Supérieure, Institut de Biologie de l´ENS, IBENS, Paris, F-75005, France; 4 Inserm, U1024, Paris, F-75005, France; 5 CNRS, UMR 8197, Paris, F-75005, France; 6 Dynamique des Structures et Interactions des Macromolécules Biologiques (DSIMB), INTS, Université Paris Diderot, Sorbonne Paris Cité, INSERM, U665, Paris, France; 7 Institut Pasteur, Plate forme Transcriptome et Epigenome, Departement Genomes et Genetique, Paris, France; 8 CNRS, UMR7238, Génomique des Microorganismes, Paris, France

**Keywords:** *Candida albicans*, Multidrug resistance, Yeast, RNA-seq, *CZF1*

## Abstract

**Background:**

Drug susceptible clinical isolates of *Candida albicans* frequently become highly tolerant to drugs during chemotherapy, with dreadful consequences to patient health. We used RNA sequencing (RNA-seq) to analyze the transcriptomes of a CDR (Candida Drug Resistance) strain and its isogenic drug sensitive counterpart.

**Results:**

RNA-seq unveiled differential expression of 228 genes including a) genes previously identified as involved in CDR, b) genes not previously associated to the CDR phenotype, and c) novel transcripts whose function as a gene is uncharacterized. In particular, we show for the first time that CDR acquisition is correlated with an overexpression of the transcription factor encoding gene *CZF1*. *CZF1* null mutants were susceptible to many drugs, independently of known multidrug resistance mechanisms. We show that *CZF1* acts as a repressor of β-glucan synthesis, thus negatively regulating cell wall integrity. Finally, our RNA-seq data allowed us to identify a new transcribed region, upstream of the *TAC1* gene, which encodes the major CDR transcriptional regulator.

**Conclusion:**

Our results open new perspectives of the role of Czf1 and of our understanding of the transcriptional and post-transcriptional mechanisms that lead to the acquisition of drug resistance in *C. albicans*, with potential for future improvements of therapeutic strategies.

## Background

The yeast *Candida albicans* is the major cause of opportunistic fungal infections in humans. In case of systemic infections, the mortality rate can reach 50% [1]. Azoles, which target the fungal P450 cytochrome 14alpha-lanosterol demethylase encoded by the *ERG11* gene, are the most commonly used antifungal molecules for candidosis treatment [2]. Unfortunately azoles only have a fungistatic effect and therefore have allowed the emergence of multidrug resistance strains in patients [3]. There are two main groups of mutations which cause azole resistance in *Candida albicans*. The first group directly targets *ERG11*, either in cis by creating *ERG11* alleles which encode a protein variant insensitive to azoles [4], or in trans by increasing the expression of *ERG11* through gain of function mutations in the *UPC2* gene, which encode a transcription factor regulating *ERG11*[5]. The second type acts by increasing the expression of membrane transporters which export the drugs and therefore decrease their intracellular concentration [5]. Two types of drug resistance, called MDR and CDR respectively, are distinguished, depending on the type of transporters which is involved. In most of the clinical isolates studied so far, the cells present either a drug resistance of the MDR or the CDR type [5,6]. Only in rare occurrences, the two kind of resistance can be observed in the same cells [6-8]. The MDR resistant cells overexpress the Mdr1 permease encoding gene, which is under the control of the Mrr1 and Cap1 transcription factors [9]. *MDR1* is homologous to the *Saccharomyces cerevisiae FLR1* transporter, which is involved in the detoxification of several drugs, including the antifungal benomyl [8,10,11]. The CDR resistant strains overexpress two ABC transporters encoding genes, *CDR1* and *CDR2*, which are homologous to the pleiotropic drug resistance transporter Pdr5 from *S. cerevisiae*[12,13]. These transporters are major determinants of the resistance to fluconazole, the most widely used azole. Their expression is controlled mainly by the Tac1 transcription factor [14]. Most of the CDR resistant strains present a loss of heterozygocity and/or aneuploidy at the *TAC1* locus, combined to gain of function mutations of *TAC1*[15,16]. Microarray and proteomic analyses identified about 30 genes which are co-overexpressed with *CDR1* and *CDR2* in all CDR clinical isolates examined [17,18]. Chromatine immunoprecipitation and *tac1Δ* strain analyses identified the *TAC1* regulon, which is composed of about ten genes mostly involved in membrane properties, including *CDR1CDR2*, the phospholipid transferase gene *PDR16*, the putative flippase gene *RTA3* or the putative sphingosine kinase gene *LCB4*[18,19].

The recently developed RNA sequencing (RNA seq) technology is revolutionizing our ability to analyse eukaryotic transcriptomes [20-22] and has given a chance to unravel the actual complexity of the CDR phenotype better. RNA seq has been proven to be more sensitive than microarrays [21]. RNA seq also results in more accurate measurements of gene expression changes [23]. Moreover, relative to standard microarray approaches, RNA seq and tiling arrays provide crude measurement and identification of transcripts, without any a priori on which region of the genome is transcribed [24,25]. Hence, two recent RNA seq and one tiling array analyses of the *Candida albicans* transcriptome identified more than 1000 new transcripts, many of which are expressed in a condition-specific way [25-27]. Most of these transcripts do not have a coding potential and may be long non coding regulatory RNAs.

In this study, we have used RNA seq to analyse the transcriptomes of a CDR strain and its isogenic drug susceptible counterpart. In addition to the genes previously shown to be associated with CDR, we could identify about 50 genes which were overexpressed in the CDR strain. In particular, we show that the transcription factor encoding gene *CZF1*, which is involved in hyphal transition and white/opaque switching, is induced together with *CDR1* and *CDR2* in Gu5. Czf1 is likely to play an important role in CDR acquisition since its overexpression is a general feature of all the CDR strains that we have tested, but was not found in MDR strains. Moreover, its deletion caused susceptibility to several unrelated drugs. Additionally, the inactivation of *CZF1* increased the resistance of the cells to cell wall perturbating agents, through the overexpression of beta glucan synthesis genes. We propose that Czf1 has a positive role on drug resistance and a negative role on cell wall integrity. Finally, we characterized a new transcribed region, previously undetected, just upstream of the *TAC1* gene, which strongly suggests that *TAC1* is subjected to complex post-transcriptional regulations, yet to be characterized. Taken together, our results open several new ways to our understanding of drug resistance acquisition in *Candida albicans* and may provide new targets for antifungal therapies.

## Results

### Transcriptional landscape of Gu4 and Gu5 strains

In order to conduct a comparative analysis of the transcriptomes of CDR versus drug susceptible *C. albicans* cells, we performed high-throughput sequencing of cDNA made from poly(A) RNAs obtained from the Gu4 and Gu5 strains. Gu4 is a fluconazole susceptible clinical isolate obtained from an early infection episode. Gu5 is the corresponding fluconazole resistant clinical isolate obtained from later episode in the same patient treated with fluconazole. Gu4 and Gu5 are therefore supposed to be isogenic and mainly differ by their resistance to fluconazole. Gu5 has been characterised as a CDR strain, showing overexpression of *CDR1* and *CDR2*, as compared with Gu4 [28]. For each strain, two biologically independent samples of RNAs were analysed by RNA seq. We obtained 27 763 991 unique mappable reads for Gu4 (96% of total reads) and 20 570 932 mappable reads for Gu5 (97% of total reads). As noticed in previous RNA seq studies, the background was very low, with no or few reads mapped in intergenic regions. The correlation coefficient between the biological replicates was very high (0.991 for Gu5 and 0.999 for Gu4). We could detect expression for 5727 (92%) of the 6177 ORFs annotated in the CGD. The 450 ORFs which were undetected in our experiments are mostly dubious ORFs or gene without clear function (71%). We arbitrarily grouped the expressed ORFs in five categories: very high expression (more than 1000 reads/nucleotides), high expression (between 100 and 1000 reads/nucleotides), medium expression (between 10 and 100 reads/nucleotides), low expression (below 10 reads/nucleotides) and very low expression (below 3 reads/nucleotides). These categories contained 3%, 20%, 57%, 12% and 8% of the genes, respectively. As described previously, the “very high expression” category was enriched in genes encoding ribosomal proteins, glycolytic enzymes, translation regulators, histones and components of respiratory complexes. The “high expression” category was enriched in genes encoding enzymes involved in various metabolisms. The “medium” and “low” expression categories did not show any significant functional enrichment.

**Figure 1:**
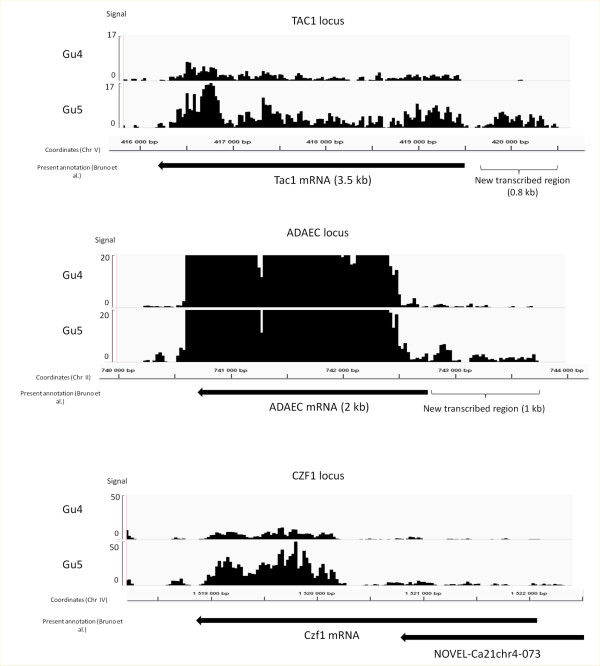
**Expression profiles obtained for *TAC1*, *ADAEC* and *CZF1.*** The density of reads along the genome has been visualized using Integrative Genome Viewer (IGV) and the CGD annotation. The signal is in reads/nucleotides. The coordinates and the transcript annotations previously available [25] are indicated below each IGV capture. *TAC1* and *ADAEC* present extension in 5’ in our data as compared with previous annotations. In the case of *TAC1*, this extension was detected only in the Gu5 strain.

We used our data to define the boundaries of each transcript, as described previously [25]. We compared the 5’ and 3’UTR predictions that we obtained with the annotation set up by a previous RNA seq analysis of the *Candida albicans* reference strain SC5314 [25]. We found no significant difference in the two annotations for more than 75% of the genes, indicating that our data is reliable and that there is no global change in 5’ and 3’UTR length between those strains. Still, we had some significant discrepancies with the annotation from Bruno et al. for about thirty genes, which showed much longer 5’ or 3’ UTR according to our data ( Additional file 1: Table S1). This is for instance the case of *TAC1ADAEC* and *CZF1* (Figure 1). These genes did not show any particular functional enrichment. Noteworthy, we did not detect significant differences in the size of transcripts between the Gu4 and Gu5 strains, with the notable and sole exception of *TAC1* which presented an extension in 5’ which was apparently specific for the azole resistant strain (Figure 1). 

Finally, we used our data to detect new transcripts. To do so, the reads from the Gu4 and Gu5 strains were summed and used to identify regions of the genome with a minimum coverage of at least 3 reads/nucleotides (with at least one read in three independent samples) for a stretch of at least 50 nucleotides. With these criteria, we identified 1130 new transcription units, 800 of which partially or totally overlapped new transcribed regions recently identified by RNA seq [25,26] or tiling arrays [27] ( Additional file 1: Table S2). We named these new transcripts NGuTs for “New Gu strains Transcripts”. The median size of NGuTs was 425 nucleotides. The majority of these NGuTs did not contain any obvious ORF and are therefore likely to be non-coding RNAs.

### Transcripts differentially expressed in the drug resistant and the drug susceptible strains

The identification of genes differentially expressed in Gu4 and Gu5 was performed combining two different statistical methods (see methods section) and using the gene annotations from the CGD, the new transcribed regions defined by previous studies [25-27] and the new transcribed regions defined in this study (see above). We found 130 genes significantly overexpressed in Gu5 and 92 genes which were underexpressed in this strain compared to Gu4 (a full list and description of these genes can be found in Additional file 1: Table S3).

### Comparison with previous microarray experiments

We first compared our results with the one obtained in a previous study, which used microarrays to analyse the transcriptomes of 4 different CDR isolates [18]. Among the 130 genes induced in Gu5, 74 (56.5%) were previously shown to be overexpressed in one or several of these CDR clinical isolates ( Additional file 1: Table S3). Among these 74 genes, 53 (72%) were found overexpressed in at least 3 of the 4 CDR strains, suggesting that most of the genes identified in our RNA seq analyses are general features of the CDR isolates. These genes include the *CDR1* and *CDR2* ABC transporter encoding genes, together with the *TAC1* gene encoding their transcriptional regulator. Remarkably, all the previously identified targets of Tac1p are found in our list, including *PDR16RTA3* and *LCB4*.

Of the 92 genes underexpressed in Gu5, 42 (46%) were previously shown to be repressed in CDR isolates. This includes genes involved in iron and metal homeostasis (*FTR1*, *SIT1*, *FET34* for instance) and amino acid metabolism (*MET3*, *OPT7*, *GAP4*, *SAM4* for instance).

### ORFs not previously associated with CDR

We identified 53 ORFs which were overexpressed in Gu5 and were not previously associated with CDR phenotype. About half of them (45%) are uncharacterized and little information is available on their function.

**Figure 2:**
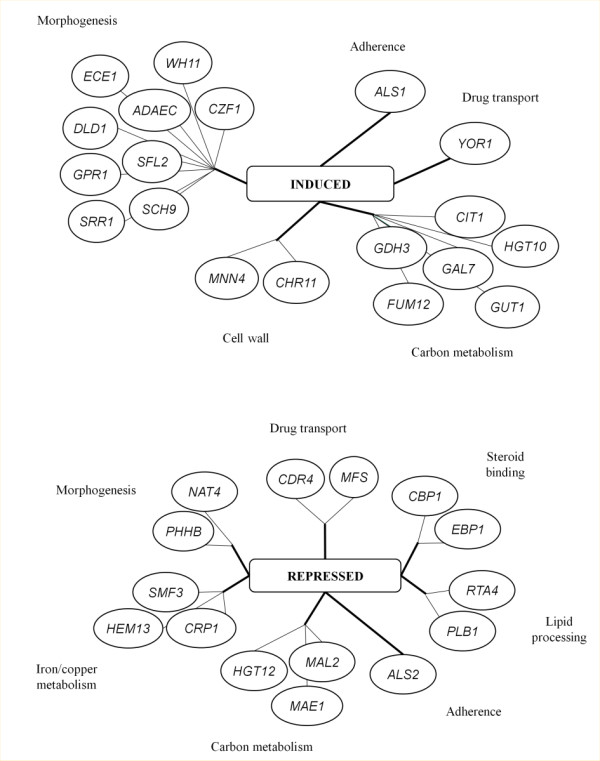
**New genes induced or repressed in the CDR isolate.** This figure represents a subset of the genes (and associated functions) which were induced or repressed in the Gu5 strain compared with Gu4 and which were not previously associated to CDR acquisition. The complete list of genes up- or down-regulated in Gu5 is available in Additional file 1: Table S3.

Among the characterized genes (Figure 2 and Additional file 1: Table S3), some of them were known as playing important roles in morphogenesis, hyphal growth and white/opaque switch. This is the case of the transcription factors Czf1 and Sfl2, the kinase Sch9, the membrane sensor Gpr1 and its target Ece1, the signal transducer Srr1, the white specific gene of unknown function ADAEC and the cytoplasmic protein Wh11. Some other induced genes are involved in cell wall maintenance and cell adhesion, like the mannosyl-transferase Mnn4 or the adhesion protein Als1. Finally, two transporters encoding genes belong to this list: Yor1, which is homologous to an oligomycin resistance gene of *S. cerevisiae* and Hgt10, which encodes a glycerol permease involved in osmotic stress response. Remarkably, four of the CDR induced genes (*CZF1*, *TAC1*, *ADAEC* and *orf19.6713*) present the particularity to have a long transcribed region upstream of the start codon according to our RNA seq data, which could be interpreted either as long 5’ UTR or as new non coding transcripts (see Figure 1 and chapter below).

Fifty genes were shown for the first time to be downregulated in Gu5. Of these, 24 (49%) are uncharacterized. Among the verified ORFs (Figure 2 and Additional file 1: Table S3), we could find the gene encoding the ABC transporter Cdr4, which is homologous to Cdr1 and Cdr2, and *RTA4*, which is homologous to the Tac1 target, *RTA3*. We also found in this group additional genes involved in copper and iron homeostasis (*HEM13CRP1* and *SMF3*) and genes encoding extracellular proteins (*PLB1ALS2MAL2*). Two genes encoding potential steroid targets, *EBP1* and *CBP1*, were also underexpressed in Gu5, which is interesting considering the previously established link between steroid exposure and CDR response [29].

**Figure 3:**
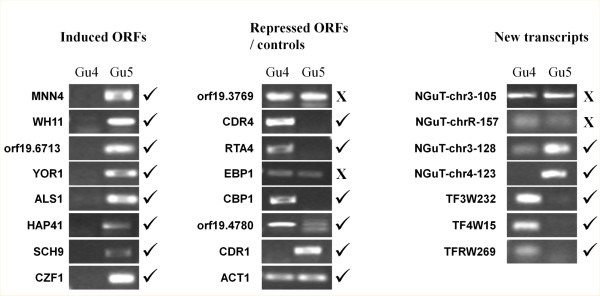
**Confirmation of RNA seq results by semi-quantitative RT-PCR.** Final point semi-quantitative RT-PCR was used to validate some of the genes found to be differentially expressed by RNA seq. *CDR1* was used as a positive control of a gene induced in the Gu5 strain. *ACT1* was used as a reference gene equally expressed in Gu5 and Gu4. The genes for which the PCR results did not confirm the RNA seq results are marked by a cross on the right of the gel picture.

To support these results, we performed semi-quantitative RT-PCR analyses for 6 repressed and 8 induced genes, using actin as a reference and *CDR1* as a positive control (Figure 3). These experiments confirmed the RNA seq results, except for *EBP1* and *orf19.3769*, for which no difference of expression was detected by this method.

### New transcripts differentially expressed in Gu5 and Gu4 strains

Among the new transcripts identified in this study, 5 were overexpressed and 8 underexpressed in Gu5 compared with Gu4. Four of the five induced transcripts were located immediately in 5’ of an induced ORF. Transcript NGuT-chr4-123 (previously identified as NOVEL-Ca21chr4-073 in [25]) was in 5’ of *CZF1* (Figure 1), NGuT-chr5-037 was in 5’ of *TAC1* (Figure 1), NGUT-chr2-038 was in 5’ of *ADAEC* (Figure 1) and NGuT-chr3-128 (previously identified as TF3W223 in [27]), was close to *orf19.6713*. The fifth transcript, NGuT-chr3-105 (NOVEL-Ca21chr3-056), is located just upstream of the *AAF1* gene, which encodes an adhesin and which is not induced in the Gu5 strain.

All the new transcripts which were repressed in Gu5 are located at more than 1 kb from the adjacent ORFs. NGUT-chrR-157 (NOVEL-Ca21chrR-103) and 160 (TFRW269) belong to the same cluster of non coding RNAs. NGUT-chr4-069 (NOVEL-Ca21chr4-040) is located in a zone rich in tRNA genes. NGUT-chrR-041 (NOVEL-Ca21chrR-026) is close to a long terminal repeat.

Semi-quantitative RT-PCR analyses for 4 repressed and 3 induced NGuTs, using actin as a reference confirmed the RNA seq results, except for NGuT-chr3-105 and NGUT-chrR-157 (Figure 3).

### New roles of the Czf1p transcription factor in multidrug resistance and cell wall maintenance

#### CZF1 deletion alters drug resistance

**Figure 4:**
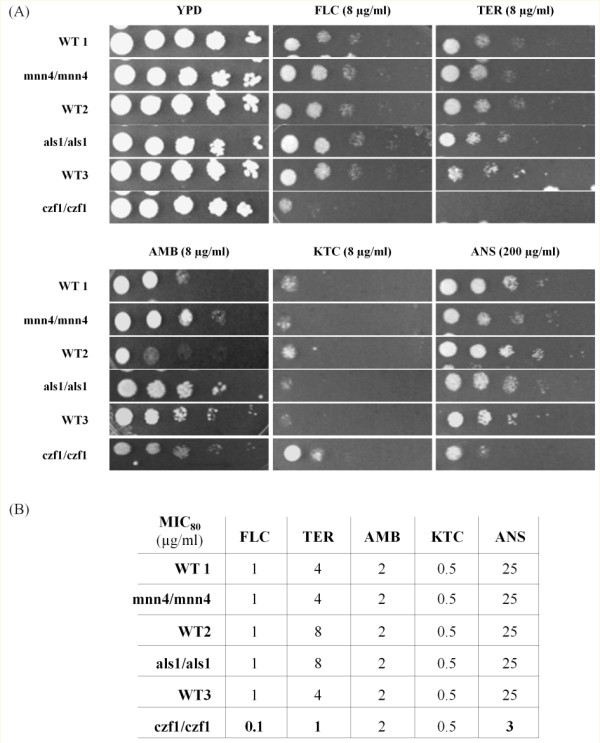
**Phenotypic profiling of some strains mutated for genes induced in Gu5.** (A). Serial dilution assays showing drugs susceptibilities of strains deleted for genes found to be induced in Gu5 and of their isogenic wild type parents. (B). MIC80 calculated for each drug and each strain. FLC, terbinamycin (TER), anisomycin (ANS), amphotericin B (AMB), and ketoconazole (KTC).

To go further in our investigation of the roles of new genes involved in the acquisition of CDR, we selected three of the induced genes, *MNN4* (for mannosylphosphorylation), *CZF1* (*C. albicans* Zinc Finger protein), and *ALS1* (Agglutinin Like Sequence), and performed drug sensitivity profiling with the corresponding null mutants (Figure 4A). Serial dilutions of cells were grown on agar plates and minimum inhibitory concentration (MIC) was determined by broth microdilution assay with different concentrations of FLC, terbinafin (TER), anisomycin (ANS), amphotericin B (AMB), and ketoconazole (KTC) (Figure 4B). The homozygous mutants for *MNN4* and *ALS1* did not exhibit any significant differences with their respective wild type strains. However, *czf1* null mutant displayed enhanced susceptibility to FLC, TER and ANS in comparison to its wild type strain (Figure 4A). MIC_80_ of the *czf1* null strain was calculated as 0.12, 1 and 3.12 μg/ml for FLC, TER, and ANS, respectively, which was ~ 3 fold less than its isogenic wild type (Figure 4B). Notably, the strain heterozygous for the deletion of *CZF1* had wild type levels of drug resistance (data not shown). *czf1* nulls did not show any differential susceptibility to AMB or KTC (Figure 4).

#### CZF1 deletion does not impair the expression of standard MDR and CDR actors

**Figure 5:**
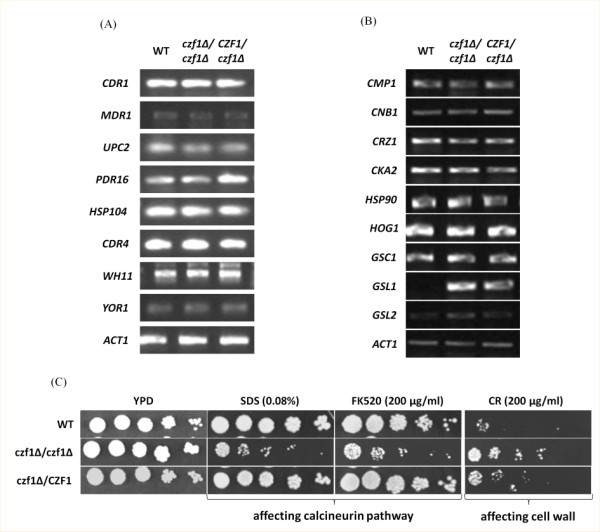
**Effects of *CZF1* deletion on genes involved in CDR, calcineurin or beta-glucan biosynthesis pathways.** The expression of several genes involved either in CDR (A) or calcineurin and beta glucan biosynthesis patways (B) was measured in *czf1Δ/czf1Δ*, *czf1Δ/CZF1* and wild type strains by final point semi-quantitative RT-PCR. *GSL1* is the only gene that seemed to be affected by the inactivation of *CZF1* in these experiments. *ACT1* was used as a reference gene. (C) growth assays testing the sensitivity of the strains mentioned above to drugs associated to SDS, FK520 and congo red.

To understand the molecular basis of the drug sensitivity described above, we measured, in the wild type and in strains mutated for one or both copies of *CZF1*, the expression levels of major actors of multidrug resistance pathways (*CDR1*, *MDR1*, *UPC2*, *PDR16*, *HSP104*) or of genes changing expression in Gu5 in our experiments (*CDR4*, *YOR1*, *WH11*). Final point semi-quantitative RT-PCR did not show any difference in the level of these genes in the wild type and in strains mutated for one or both copies of *CZF1*, indicating that Czf1 does not act on the steady state expression of MDR and CDR actors in a laboratory strain (Figure 5A).

#### CZF1 negatively controls beta-glucan synthesis and resistance to cell wall perturbation agents

The sensitivity of the *czf1* null mutant to various drugs prompted us to test the phenotype of this mutant in presence of drugs targeting the calcineurin pathway or cell wall integrity. Calcineurin has been shown to play multiple roles in stress responses including resistance to FLC and to membrane perturbation [30,31]. It has also been shown to influence the dimorphic switch, a process which is controlled by Czf1 [31]. We tested growth of wild type, *czf1Δ/czf1Δ* or *czf1Δ/CZF1* strains in presence of the membrane perturbation agent SDS and the specific calcineurin inhibitor FK506. We observed that the double mutant was more sensitive to these drugs than the single mutant and the wild type strain (Figure 5C). We tested the expression of several direct or indirect actors of the calcineurin pathway (*CMP1CNB1CRZ1HSP90* and *HOG1).* We did not detect any effect of the partial or total inactivation of *CZF1* on the expression of these genes (Figure 5B). Reciprocally, we tested the expression of *CZF1* in various strains mutated for one or several actors of the calcineurin pathway. Again, we did not see any effects of the inactivation of the calcineurin pathway on the steady-state expression of *CZF1* (data not shown).

In *C. albicans*, the cell wall plays important roles in virulence, defense against host and resistance to some drugs [32]. Surprisingly, we observed that the *czf1Δ/czf1Δ* strain was more resistant than the wild type to congo red (a cell wall perturbation agent) (Figure 5C). The heterozygous mutant exhibited an intermediate phenotype (haploinsufficiency), suggesting a dosage effect for the role of *CZF1* in cell wall maintenance. Congo red is considered to act on the cell wall glucan structure. Therefore, we measured the expression of the three beta 1,3-glucan synthase encoding genes (*GSC1GSL1* and *GSL2*) in presence and absence of *CZF1* (Figure 5B). We observed no or few differences in *GSC1* and *GSL2* expression. In contrast, we observed a clear overexpression of *GSL1* in the strains deleted for one or two copies of *CZF1*, compared to the wild type. This result suggests that Czf1 negatively controls the expression of this gene and that the resistance to congo red that we observed in the *czf1* mutants could be linked to the partial deregulation of beta-glucan synthesis. Interestingly, a defect in beta-glucan activity has been shown to cause sensitivity to many different drugs, including azoles [33]. However, the expression of *GSL1GSC1* and *GSL2*, tested by RNA-seq and semi-quantitative RT-PCR, was unchanged in the Gu5 strain as compared with Gu4 (data not shown).

#### CZF1 overexpression is commonly associated with CDR acquisition

**Figure 6:**
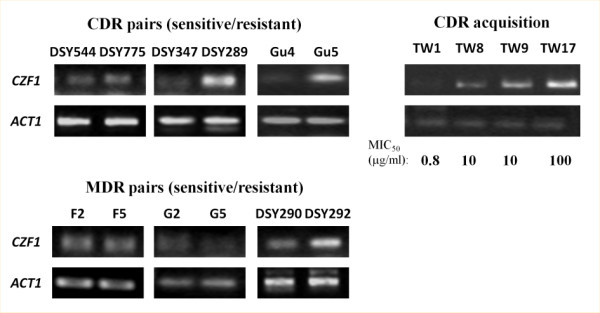
***CZF1* induction is commonly associated with CDR acquisition.** The expression of *CZF1* was measured by final point semi-quantitative RT-PCR in different pairs of drug sensitive/resistant strains presenting either a drug resistance of the CDR or of the MDR kind. On the right panel, the expression of *CZF1* was measured in a series of isolates coming from the same patient at different step of fluconazole resistance acquisition [34]. Seventeen isolates were available from the original publication, four of them (numbers 1, 8, 9 and 17) were tested here. The MIC_50_ of each isolate for fluconazole is indicated. *ACT1* was used as a reference gene.

To check whether an upregulation of *CZF1* is a general phenomenon among drug resistant clinical isolates, we examined the expression of *CZF1* in six azole sensitive (AS) and azole resistant (AR) matched pairs (Figure 6). The first group of AS/AR isolates includes strains (DSY347/DSY289; DSY544/DSY775 and Gu4/Gu5) in which the AR isolate is characterized by the overexpression of *CDR1* and *CDR2* (CDR group). The second group included strains (F2/F5; G2/G5 and DSY290/DSY292) where AR isolates showed an overexpression of *MDR1* (MDR group). Our quantitative RT-PCR data revealed that *CZF1* was upregulated in two AR isolates of the CDR group and one of the MDR group (DSY290/DSY292; DSY347/DSY289; Gu4/Gu5) (Figure 6, upper left). In contrast, the levels of *CZF1* remained unchanged in the other pairs of isolates (Figure 6, bottom left). We next analyzed the expression of *CZF1* in a series of clinical isolates acquired from a single HIV-infected patient over the period of two years where the levels of FLC resistance of the strain increased over 200-fold [34] (Figure 6, right panel). We observed a gradual overexpression of *CZF1,* which followed the increase in FLC resistance of those isolates (Figure 6, right panel). 

Taken together these results strongly suggest that the overexpression of *CZF1* is generally correlated to the acquisition of the CDR phenotype, with the notable exception of the DSY544/DSY775 pair.

## Discussion

In this work, we have used RNA-seq to analyze the transcriptome changes associated with CDR acquisition in a pair of clinical isolates in the human pathogen *C. albicans*. Our aim was to obtain an accuracy, sensitivity and completion unreached by previous microarray analyses of the same strains. Based on previous RNA-seq studies, we expected a better detection of mRNAs and of their expression changes, the identification of unannotated transcripts possibly up or down regulated in the CDR strain, and the detection of changes in the 5’ and 3’ UTR lengths compared with available annotations. Indeed, our data pointed out about one hundred genes, which were either induced or repressed in the Gu5 strain compared with Gu4, and which were not associated to CDR acquisition by previous studies. Although we identified more than one thousand new transcripts (some previously annotated by RNA seq or tiling arrays, some completely new), very few of them changed expression in the CDR strain. Four of these new transcripts were found to be induced in Gu5, but their position just upstream of annotated genes, which are similarly induced in Gu5, raises doubts on their actual existence as independent transcripts, or as long, previously undetected, 5’ UTR of the downstream gene.

The analyses of this large amount of data led to two important conclusions, which open new perspectives for our understanding of multidrug resistance in this pathogenic yeast. First, we revised the annotation of the *TAC1* genomic region, identifying a new transcribed region in 5’ of the *TAC1* mRNA. Second, we suggested new roles for the Czf1 transcription factor in CDR acquisition and cell wall maintenance.

### A new transcribed region upstream of TAC1 and its potential impact on TAC1 expression regulation

RNA seq and tiling arrays experiments have allowed the systematic annotation of transcript boundaries in three yeast species: *S. cerevisiaeC. albicans* and *C. parapsilosis*[21,25,27,35]. In all three species, the 5’ and 3’UTR have been shown to be generally short, with an average size ranging between 50 and 80 nucleotides. However, in all these species, many exceptions have been described. In *C. albicans*, about 100 genes have 5’ UTR longer than 500 bp [27]. Interestingly, this group is enriched in genes encoding transcriptional regulators and genes involved in filamentation. These include the *CZF1* transcription factor encoding gene, which was induced in our experiments and which has a 5’UTR of about 2 kb ([27,36]; this study). This observation is also true in mammals in which many genes with long 5’UTR encode regulators and proto-oncogenes [37]. Long 5’UTR usually have important roles in the post-transcriptional and translational regulation of gene expression. In the yeast *Saccharomyces cerevisiae*, the expression of several transcription factors, including Gcn4 (amino acid starvation), Yap1 (oxidative stress response) and Yap2 (cadmium response), is regulated by the presence of short upstream ORFs (uORFs) in their long 5’UTR region, which exert negative or positive actions on the translation of the main ORF or on mRNA stability, depending on the cellular context [38]. Recent studies have identified about 250 genes containing potential uORFs in this species [21].

**Figure 7:**
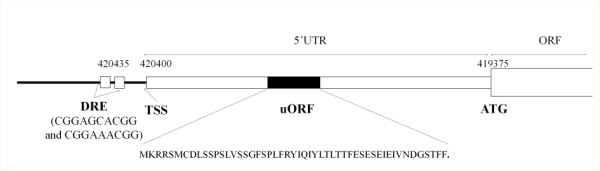
**New annotation of the 5’ region of *TAC1*.** The positions of the start codon (ATG), the transcription start site, the potential uORF in the 5’UTR (together with its sequence) and the two Tac1 binding sites (DRE) are indicated.

As mentioned in the introduction, *TAC1* encodes the major transcriptional regulator of CDR genes. Its own mRNA expression is co-induced with those of its main targets, *CDR1* and *CDR2*, possibly due to an autoregulatory loop [18,29]. In previous transcript annotations, *TAC1* mRNA was described as having a 5’UTR of about 300 nt [25,27]. Our RNA seq data detected a transcribed region up to 1 kb upstream of the *TAC1* start codon, which was apparently specific of the Gu5 strain (Figure 1). This could be interpreted as a new transcript expressed only in the CDR strain, as an alternative transcription start site (TSS) specific for Gu5 (alternative TSS had been described in *C. albicans*, for instance in the case of the *EFG1* transcription factor encoding gene [39]) or as the normal TSS of *TAC1* which could not be detected in drug sensitive strains because expression of *TAC1* in those strains and RNA-seq coverage in previous experiments and in this study were too low. 5’RACE and northern blot experiments could resolve if it is an alternative TSS or a new transcript. However, the strong correlation between the expression of the two regions (Figures 1 and 3) suggests that *TAC1* mRNA presents, at least in the Gu5 strain, an unusually long 5’UTR, which was previously not detected. Considering the important roles of long 5’UTR in translation regulation, this opens exciting new perspectives to the post-transcriptional regulation of *TAC1* expression, which was poorly investigated until now. Especially, we observed in this 5’UTR the presence of one large (about 50 codons) uORF in the same orientation and phase than the *TAC1* ORF (Figure 7), which suggests the existence of regulatory mechanisms similar to the ones described for *GCN4YAP1* or *YAP2* in *S. cerevisiae*. The analysis of ribosome occupancy at *TAC1* mRNA in CDR matched pairs, either measured by global ribosome profiling or by polysomes gradients, will give information on this potential regulation. Moreover, a long 5’UTR would have important implications on the way we consider the transcriptional regulation of *TAC1*. Indeed, yeast enhancers are usually located close upstream to the TSS. Since 5’UTR regions are also limited in size, people tended to look for DNA consensus motifs within 800 bp upstream of the start codon of the genes. The fact that the actual TSS of *TAC1* would be located about 0.8 kb upstream of all previous estimations also change the potential position of its promoter region. For instance, it was shown that Tac1 binds its own promoter, which led to the hypothesis that it is regulated through a positive auto-regulatory loop [18]. However, this hypothesis was challenged by the fact that the two potential Tac1 binding motifs (DRE) in this region were located more than 1 kb upstream of *TAC1* ATG. In the annotation proposed in Figure 7, these DRE would be located very close to the TSS, which strongly supports their functional role in the activation of *TAC1* expression. 

### New roles for Czf1 in CDR acquisition and cell wall maintenance

Czf1 is a zinc finger transcription factor mostly known for its role in the epigenetic white/opaque switch, which controls cell and colony morphogenesis and properties [40]. Czf1 acts synergistically to Wor1 and Wor2 transcription factors, and antagonistically to Efg1, to promote the opaque state. Czf1 also has a role in hyphal growth, (also called filamentation). Czf1 mutants filament normally under standard filamentation induction conditions, but they are defective in hyphal development in anaerobic conditions [41]. This phenotype is dependent on the composition of the media, for instance in terms of carbon source. The positive role of Czf1 on hypoxic filamentation is under the negative control of the TOR pathway and more specifically, of the Sch9 kinase [42]. As for the white/opaque switch, Efg1 plays an important and complex role in filamentation. In aerobic conditions, it is a positive regulator of hyphal development, whereas in hypoxia, it counteracts the positive action of Czf1 on this process [43]. Direct cross-talks exist between Efg1 and Czf1: Efg1 binds to the promoter of *CZF1* and repress its expression, while Czf1 has a similar effect on the *EFG1* promoter [36,44]. More recently, Czf1 has been shown to be required for wild type adherence to silicone devices [45]. Despite these roles in morphogenesis, the list of Czf1 targets has not been established to date. A partial transcriptome analysis of the *czf1* mutant, conducted on 250 genes using the Nanochip technology, detected very few expression changes in this mutant [45].

Our data strongly suggest that Czf1 plays a role in the acquisition of multidrug resistance of the CDR type. First, *CZF1* is overexpressed in all CDR mutants that we tested. Noteworthy, some other actors of white/opaque switch and hyphal growth were also overexpressed in the Gu5 strain, including *SCH9*, *ADAEC*, *WH11* or *SFL2*. Second, the *czf1* null mutant is sensitive to many unrelated drugs, including azoles and molecules targeting the calcineurin pathway. Remarkably, *CZF1* does not seem to be involved in drug resistance phenotypes of the MDR kind, since its expression was unchanged in MDR mutant strains. The drug sensitivity associated with the inactivation of Czf1 does not seem to be due to standard drug resistance pathways, since the level of expression of the main actors of the CDR, MDR and calcineurin pathways were unchanged in this mutant. However, the fact that Czf1 does not control the steady-state expression levels of those genes does not mean that it is not involved in their induction following drug exposure. This hypothesis will be tested by inactivating *CZF1* in the Gu5 and Gu4 backgrounds and testing the effect of this inactivation on drug resistance and gene expression.

Surprisingly, we observed that the *czf1* mutant was resistant to congo red, a cell wall perturbation agent which targets beta-glucans, the most abundant component of *Candida albicans* cell wall. This phenotype was haploinsufficient, since the heterozygous mutant exhibited an intermediate resistance between the null mutant and the wild type. Congo red is thought to target the glucan architecture by directly interacting with glucan chains, therefore inhibiting synthesis of glucan polymers [46]. We observed that the *GSL1* gene encoding one of the three beta 1,3 glucan synthase subunits, was strongly overexpressed in the *czf1* mutant. This suggests that Czf1 has a negative effect on the expression of some of the actors of the glucan synthesis pathway, which could explain the congo red resistance observed in the null mutant. Many functional connections have been established between cell wall components and hyphal growth. *GSL1* itself has been shown to be down-regulated during filamentation [47], a process that is positively regulated by Czf1. Notably, Efg1 is also involved in cell wall architecture and *efg1* mutants show a haploinsufficiency phenotype [47,48]. The deletion of one or two copies of *EFG1* leads to a decrease in the expression of many enzymes involved in cell wall biogenesis, including beta1-3 glucan synthases. This causes important changes in cell wall thickness and composition [48]. Again, Czf1 and Efg1 seem to antagonize each other in the regulation of cell wall maintenance. The over-expression of some cell wall protein encoding genes has been extensively described as a compensatory mechanism in response to impairments in cell wall biosynthesis or to cell wall damages, both in *S. cerevisiae* and *C. albicans* (see for instance [49-51]). This phenomenon is largely controlled by the cell wall integrity (CWI) pathway [52] but part of it is independent of CWI [53]. For instance, treatment with caspofungin (which directly inhibits the activity of the essential beta-glucanase subunit encoded by *GSC1*) triggers a compensatory process which leads to the overexpression of *GSL1*, similarly to what was observed in the czf1 null mutant [51]. We suggest that Czf1 could play a role in this process.

## Conclusions

**Figure 8:**
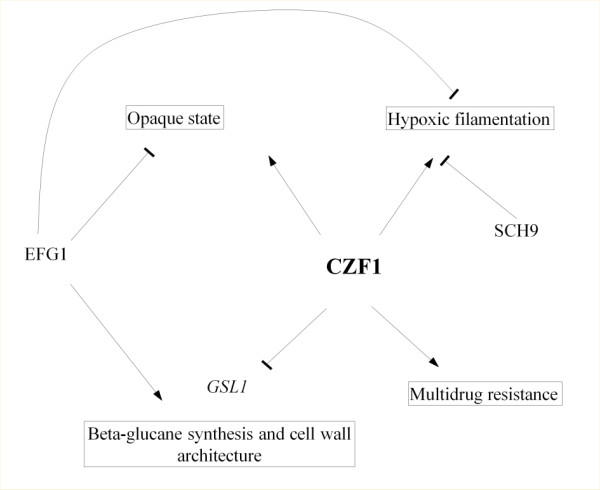
**New roles for Czf1**. Czf1 had been shown to antagonize the role of Efg1 in hypoxic filamentation and white/opaque switching. In this study, we suggest new roles for Czf1 in MDR and in cell wall architecture. In this last role, Czf1 would antagonize the positive role of Efg1 on the expression of genes involved in cell wall biosynthesis and maintenance, as for instance in the case of *GSL1*.

In conclusion, our results led us to propose two new roles for Czf1 in drug resistance and cell wall maintenance (Figure 8). The connection between these two roles is unclear. Several cell wall proteins are induced in CDR strains (*CRH11ALS1SCW11PGA13AGP2IFF4RBT5EXG2ECM331CSP37*, etc.) ([18]; this study). However, some CDR strains have cell wall defects, while others (including Gu5) seem to have normal cell wall architectures (Singh and Prasad, submitted). As mentioned above, Gsc1 (previously known as Fks1) is the main target of antifungals of the echinocandin family (which includes caspofungin) and mutations in *GSC1* confer resistance to these drugs [54,55]. However, neither *GSL1* nor *GSC1* show any significant changes in the CDR strains and the *GSL1* overexpression in the *czf1* null mutant does not explain the large spectrum of drug sensitivity observed for this strain. The systematic determination of the Czf1 targets by transcriptomic analyses of the *czf1* mutant and chromatine immunoprecipitation will certainly thrfow some light on its role in these two processes. 

## Methods

### Material

All the media components were obtained from HiMedia (Mumbai, India). Amphotericin B (AMB), terbinafin, anisomycin, ketoconazole, sodium dodecyl sulphate, FK520, congo red were purchased from Sigma chemicals Co. (St. Louis, MO). Ranbaxy India generously provided FLC. Reverse transcriptase kit was purchased from fermentas, India. Caspofungin was generously provided by Dr. Neeraj Chauhan (Public Health Research Institute and Department of Microbiology and Molecular Genetics, New Jersey, USA). dATP, dGTP, dTTP and dCTP were bought from Sigma chemicals Co. (St. Louis, MO).

### Yeast strains and growth conditions

Gu4 and Gu5 strains were described elsewhere [28]. The strains of *Candida albicans* mutated in various genes (*ALS1MNN4CZF1*) that were used for phenotypic tests, were obtained from Dr. Ibrahim, A.S. (David Geffen School of Medicine at UCLA Los Angeles Biomedical Research Institute), Dr. Gow, N. (The university of Aberdeen, Scotland) and Dr. Kumamoto, C.A. (Tufts University, Boston, USA) respectively. The complete list of strains can be found in Additional file 1: Table S4. Cells were grown in YPD rich medium (2% glucose, 1% bactopeptone, 1% yeast extract) at 30°C for 48 hrs.

### RNA extraction

Twenty ml of exponentially growing (OD = 0.6-0.7) Gu4 or Gu5 cells were flash frozen in 30 ml of cold ethanol, centrifuged for 5 minutes at 3000 g, washed with sterile water and centrifuged again. The cell pellets were stored at −80°C. RNA were extracted using the RNeasy kit (Qiagen), with DNAse treatment. The RNA quality was checked on a bioanalyzer Nanochip (Agilent).

### RNA sequencing

#### cDNA libraries and sequencing

Strand non-specific cDNA libraries (mRNA-Seq 8-Sample Prep Kit) were prepared according to manufacturer’s instructions (Illumina). The validation of libraries was made with Bioanalyser DNA 1000 chip (Agilent). All libraries were sequenced in single read mode, using a Genome Analyser instrument (Illumina).

#### Reads mapping and counting

Before read mapping, we trimmed polyN read tails, removed reads with a length lower or equals to 11 bases and discarded reads with quality mean lower or equals to 12. We aligned from the 4 samples a total of 48334923 bp single end reads produced from the Illumina GAII device using the SOAP2 software (version 2.20) [56] on the *Candida albicans* genome (assembly 21). The mapping tool was configured to return all alignment hits by setting the seed length to 28 bp. In addition we authorized with SOAP2 up to 5 mismatches with 2 in the seed.

Before expression estimation, alignments from reads that matches more than one time on the reference genome were removed. In addition, we searched for new transcripts by combining the Gu4 and Gu5 reads and identifying regions of the genome with a minimum coverage of at least 3 reads/nucleotides (with at least one read in three independent samples) for a stretch of at least 100 nucleotides. To compute gene expression abundance, we used *Candida albicans* GFF genome annotation from assembly 21 together with the annotation file for new transcripts and we counted all overlapping regions between alignments and listed exons. The complete results have been deposited at the GEO database (accession number: GSE38298).

#### Data mining

Functional analyses of the genome-wide data were conducted using the CGD GO (gene ontology) term finder [57], with default parameters.

#### Differential analyses

Genes differentially expressed between Gu5 and Gu4 strains were identified applying two different methodologies available in the R programming language, *i.e.* DESeq [58] and edgeR [59,60]. Default parameters were used and genes with p-value lower than 0.01 with the two methods were finally selected for further analysis ( Additional file 1: Table S3).

### Final point semi-quantitative RT-PCR analyses

RT-PCR was done using the RevertAidTM H Minus kit (MBI, Fermentas). Briefly, 1 μg isolated RNA was primed with oligo (dT)18 for cDNA synthesis at 42°C for 60 min. Reverse transcription reaction was terminated by heating at 70°C for 5 min. The synthesized cDNA product (2 μl) was directly used for PCR amplification reaction (50 μl) using gene specific forward and reverse primers. The amplified products were gel electrophoresed and quantitated by using Quantity One software by Bio Rad gel documentation system. The RNA samples used for these validation experiments were independent from the samples used for RNA seq. The sequences of the primers used for PCR reactions can be found in Additional file 1: Table S5.

### Phenotypic tests

Drug-susceptibility was tested by broth-microdilution assay according to CLSI (Clinical and Laboratory Standards Institute) and serial dilution assay essentially as described previously (CLSI, 2008). Following drugs stocks (in parenthesis) and working concentration were used 2.5 μg/ml of FLC (5 mg/ml), 4 μg/ml of TER (4 mg/ml), 4 μg/ml of AMB (4 mg/ml), 150 μg/ml of ANS (20 mg/ml), 0.5 μg/ml of KTC (1 mg/ml), 50 ng/ml of CAS (1 mg/ml), 0.08% SDS, 200 μg/ml of CR (10 mg/ml), and 200 μg/ml FK520 (20 mg/ml).

## Competing interests

The authors declare that they have no competing interests.

## Authors’ contributions

SD made all the validation experiments of RNA seq data, phenotypic analyses of mutant strains, and RT-PCR, and contributed to the manuscript. MB, GL, SL and SLC performed the bioinformatic and statistical analyses of the RNA seq data, designed the browser to visualize these data and format them for submission to GEO. OS and JYC performed the RNA seq experiments. RP conceived the project, interpreted the data and contributed to the manuscript. FD conceived the project, prepared the RNA samples for RNA seq, analyzed and interpreted the RNA seq data and wrote the manuscript. All authors read and approved the final manuscript.

## Supplementary Material

Additional file 1 **Table S1.** Genes with enlarged 3' or 5' UTR boundaries as compared with the annotation of Bruno et al. 2010. **Table S2.** annotation and coordinates of new transcripts found based on our RNA seq data. Overlap with previously annotated transcripts are indicated (annotations taken from Candida Genome Database, Bruno et al., 2010; Sellam et al., 2010 and Tuch et al., 2010). **Table S3.** CDR Induced genes. Genes that were found significantly overexpressed in Gu5 by Deseq and EdgeR. Log2(ratios) are indicated, together with CGD descriptions. The CDR isolates column indicate the number of different isolates in which the gene was previously found to be overexpressed, based on the studies of Liu et al., 2007 and Znaidi et al., 2006. **Table S4.** List of the strains used in the study. **Table S5.** Sequences of the primers used in the study. Click here for file
