# Supplementary material for: RNA sequencing revealed novel actors of the acquisition of drug resistance in Candida albicans
Source: BMC Genomics. 2012 Aug 16;13:396. doi: 10.1186/1471-2164-13-396 (PMC3447688; doi:10.1186/1471-2164-13-396)
Supplement: Additional file 1 — Table S1. Genes with enlarged 3' or 5' UTR boundaries as compared with the annotation of Bruno et al. 2010. Table S2. annotation and coordinates of new transcripts found based on our RNA seq data. Overlap with previously annotated transcripts are indicated (annotations taken from Candida Genome Database, Bruno et al., 2010; Sellam et al., 2010 and Tuch et al., 2010). Table S3. CDR Induced genes. Genes that were found significantly overexpressed in Gu5 by Deseq and EdgeR. Log2(ratios) are indicated, together with CGD descriptions. The CDR isolates column indicate the number of different isolates in which the gene was previously found to be overexpressed, based on the studies of Liu et al., 2007 and Znaidi et al., 2006. Table S4. List of the strains used in the study. Table S5. Sequences of the primers used in the study. [file 1471-2164-13-396-S1.pdf]

Supplementary table S1: Genes with enlarged 3' or 5' UTR boundaries as compared with the annotation of Bruno et al., 2010.

ORF	Gene Name	Difference with previous annotations (nt)	UTR	Description
orf19.2631		1240	5'	Hap43p-induced gene
orf19.4282	IFH1	1146	3'	Transcription factor that forms a heterodimer with Fhl11p that is tethered to promoters by Tbf1p; positively regulates rRNA and ribosomal protein gene transcription
orf19.868	**ADAEC**	989	5'	Protein of unknown function; transcription is specific to white cell type
orf19.5462		971	3'	
orf19.5762	PGA61	833	3'	Putative GPI-anchored protein
orf19.405	VCX1	773	3'	Putative H+/Ca2+ antiporter; fungal-specific (no human or murine homolog)
orf19.3188	**TAC1**	725	5'	Transcriptional activator of drug-responsive genes including CDR1 and CDR2; has Zn(2)-Cys(6) binuclear cluster; binds DRE element; gene in zinc cluster region near MTL locus; resequencing indicates that TAC1 spans orf19.3188 and orf19.3189
orf19.5491		658	3'	Ortholog of *C. parapsilosis CDC317* : CPAR2_104720
orf19.727		646	3'	Predicted ORF in Assemblies 19, 20 and 21; member of a family encoded by FGR6-related genes in the RB2 repeat sequence
orf19.1171		604	3'	Ortholog of *C. parapsilosis CDC317* : CPAR2_208080
orf19.2397.3		598	5'	Putative aminotransferase; Hap43p-repressed gene; homozygous transposon insertion causes decreased colony wrinkling under filamentous growth-inducing conditions, but does not block true hyphal formation in liquid media
orf19.5607		577	5'	Ortholog of *C. parapsilosis CDC317* : CPAR2_601140
orf19.3127	**CZF1**	557	5'	Transcriptional regulator of white-opaque switching frequency; hyphal growth regulator; expression in S. cerevisiae causes dominant-negative inhibition of pheromone response; required for yeast cell adherence to silicone substrate

orf19.7016	PHM5	530	3'	Ortholog(s) have endopolyphosphatase activity, exopolyphosphatase activity, role in polyphosphate catabolic process and fungal-type vacuole membrane, nucleus localization
orf19.7360		510	3'	Ortholog of *C. parapsilosis CDC317* : CPAR2_807220
orf19.4055		508	5'	Protein similar to S. cerevisiae Ybr075wp; transposon mutation affects filamentous growth; clade-associated gene expression
orf19.7342	AXL1	507	5'	Putative endoprotease; induced by alpha factor; transcription is upregulated in an RHE model of oral candidiasis and in clinical isolates from HIV+ patients with oral candidiasis
orf19.6273	SDH2	500	3'	Succinate dehydrogenase, Fe-S subunit; localizes to surface of yeast cells, but not hyphae; transcriptionally regulated by iron; expression greater in high iron; repressed by nitric oxide, Hap43p; induced during log phase aerobic growth
orf19.637		499	3'	Ortholog of *C. glabrata CBS138* : CAGL0J06666g
orf19.3887		493	3'	Ortholog of *C. glabrata CBS138* : CAGL0J06666g
orf19.6927	PEP8	492	3'	Protein similar to S. cerevisiae Pep8p, which is involved in retrograde transport; transposon mutation affects filamentous growth
orf19.1105.2	PGA56	490	3'	Regulator of sorbose utilization; putative GPI-anchor; predicted helix-loop helix domain; hyphal induced; induced during cell wall regeneration; colony morphology-related gene regulation by Ssn6p; transcription activated by Tbf1p, Hap43p
orf19.5700	TLO11	473	5'	Member of a family of telomere-proximal genes of unknown function; may be spliced in vivo
orf19.5700	TLO11	463	3'	Member of a family of telomere-proximal genes of unknown function; may be spliced in vivo
orf19.4361	IFF3	455	3'	Putative GPI-anchored protein

orf19.1470	RPS26A	454	5'	Predicted ribosomal protein; regulated by Nrg1p, Tup1p; genes encoding cytoplasmic ribosomal subunits, translation factors and tRNA synthetases are downregulated upon phagocytosis by murine macrophage; alternatively spliced intron in 5'-UTR
orf19.1543	OPI1	450	3'	Functional homolog of S. cerevisiae Opi1p, which is a transcriptional repressor of INO1 involved in inositol biosynthesis; has leucine zipper and putative Opi1-Sin3 interaction domain; interacts with ScSin3p, but not CaSin3p
orf19.31		450	5'	
orf19.4365		444	5'	Has domain(s) with predicted RNA methyltransferase activity and role in RNA processing
orf19.997		436	5'	Plasma membrane-associated protein of unknown function; 1 predicted transmembrane domain
orf19.5206		436	3'	Ortholog(s) have unfolded protein binding activity, role in box H/ACA snoRNP assembly and cytosol, nucleoplasm localization
orf19.5932		430	3'	Ortholog(s) have fungal-type vacuole membrane localization
orf19.6897		425	3'	
orf19.1186		415	3'	
orf19.854	UGA11	407	3'	Putative gamma-aminobutyrate (GABA) transaminase; macrophage-induced gene; overlaps orf19.854.1, which is a region annotated as a blocked reading frame

Supplementary table S2: annotation and coordinates of new transcripts found based on our RNA seq data. Overlap with previously annotated transcripts are indicated (annotations taken from Candida Genome Database, Bruno et al., 2010; Sellam et al., 2010 and Tuch et al., 2010)

Transcript_Name	Chromosome	Exon_Lefts	Exon_Rights	overlapping previously annotated transcripts
NGUT-chr1-001	Ca21chr1	875	1075	
NGUT-chr1-002	Ca21chr1	1125	1300	
NGUT-chr1-003	Ca21chr1	1350	1525	ntar2
NGUT-chr1-004	Ca21chr1	1625	2325	NOVEL-Ca21chr1-001
NGUT-chr1-005	Ca21chr1	2425	2550	
NGUT-chr1-006	Ca21chr1	2625	2700	
NGUT-chr1-007	Ca21chr1	2825	2925	
NGUT-chr1-008	Ca21chr1	4400	4450	
NGUT-chr1-009	Ca21chr1	4500	4575	
NGUT-chr1-010	Ca21chr1	4875	5050	
NGUT-chr1-011	Ca21chr1	8725	8775	
NGUT-chr1-012	Ca21chr1	9675	9775	
NGUT-chr1-013	Ca21chr1	9825	10250	NOVEL-Ca21chr1-004
NGUT-chr1-014	Ca21chr1	35800	35925	
NGUT-chr1-015	Ca21chr1	41900	42075	
NGUT-chr1-016	Ca21chr1	46120	46725	ntar9
NGUT-chr1-017	Ca21chr1	57800	58325	NOVEL-Ca21chr1-005
NGUT-chr1-018	Ca21chr1	79925	80025	
NGUT-chr1-019	Ca21chr1	112325	112525	
NGUT-chr1-020	Ca21chr1	135050	135225	
NGUT-chr1-021	Ca21chr1	137450	138000	ntar14
NGUT-chr1-022	Ca21chr1	138525	139825	ntar15
NGUT-chr1-023	Ca21chr1	148075	148550	NOVEL-Ca21chr1-008
NGUT-chr1-024	Ca21chr1	149100	149465	NOVEL-Ca21chr1-009
NGUT-chr1-028	Ca21chr1	194000	194375	
NGUT-chr1-029	Ca21chr1	207925	208075	
NGUT-chr1-030	Ca21chr1	229925	230350	TF1C333
NGUT-chr1-031	Ca21chr1	230575	231625	NOVEL-Ca21chr1-010
NGUT-chr1-032	Ca21chr1	231520	231825	
NGUT-chr1-033	Ca21chr1	232000	232325	
NGUT-chr1-034	Ca21chr1	247750	248275	NOVEL-Ca21chr1-011
NGUT-chr1-035	Ca21chr1	281675	282100	NOVEL-Ca21chr1-012
NGUT-chr1-036	Ca21chr1	291025	291650	
NGUT-chr1-037	Ca21chr1	327650	328300	NOVEL-Ca21chr1-013
NGUT-chr1-038	Ca21chr1	344975	345225	NOVEL-Ca21chr1-014
NGUT-chr1-039	Ca21chr1	349250	350375	NOVEL-Ca21chr1-015
NGUT-chr1-040	Ca21chr1	353525	353900	NOVEL-Ca21chr1-016
NGUT-chr1-041	Ca21chr1	404150	405000	NOVEL-Ca21chr1-018
NGUT-chr1-042	Ca21chr1	408600	409150	NOVEL-Ca21chr1-019
NGUT-chr1-043	Ca21chr1	426950	427275	
NGUT-chr1-044	Ca21chr1	444800	445150	
NGUT-chr1-045	Ca21chr1	445575	446400	
NGUT-chr1-046	Ca21chr1	446775	447500	NOVEL-Ca21chr1-020

NGUT-chr1-047	Ca21chr1	447650	448300	
NGUT-chr1-048	Ca21chr1	464200	464475	
NGUT-chr1-049	Ca21chr1	472375	472875	NOVEL-Ca21chr1-022
NGUT-chr1-050	Ca21chr1	472975	474000	NOVEL-Ca21chr1-023
NGUT-chr1-051	Ca21chr1	481950	482100	
NGUT-chr1-052	Ca21chr1	488600	488850	NOVEL-Ca21chr1-024
NGUT-chr1-053	Ca21chr1	512700	513025	NOVEL-Ca21chr1-025
NGUT-chr1-054	Ca21chr1	514775	515375	
NGUT-chr1-055	Ca21chr1	536675	537000	
NGUT-chr1-056	Ca21chr1	564775	565150	
NGUT-chr1-057	Ca21chr1	595775	596675	NOVEL-Ca21chr1-027
NGUT-chr1-058	Ca21chr1	676275	676875	NOVEL-Ca21chr1-029
NGUT-chr1-059	Ca21chr1	700425	701200	NOVEL-Ca21chr1-030
NGUT-chr1-060	Ca21chr1	740050	740375	NOVEL-Ca21chr1-032
NGUT-chr1-061	Ca21chr1	757950	758250	
NGUT-chr1-062	Ca21chr1	794350	794875	NOVEL-Ca21chr1-033
NGUT-chr1-063	Ca21chr1	804925	805775	NOVEL-Ca21chr1-034
NGUT-chr1-064	Ca21chr1	812675	812950	ntar80
NGUT-chr1-065	Ca21chr1	844275	844600	
NGUT-chr1-066	Ca21chr1	860850	861525	NOVEL-Ca21chr1-036/ntar83
NGUT-chr1-067	Ca21chr1	861575	862000	ntar84
NGUT-chr1-068	Ca21chr1	874075	874425	ntar86
NGUT-chr1-069	Ca21chr1	878300	878375	TF1W156
NGUT-chr1-070	Ca21chr1	888450	889325	NOVEL-Ca21chr1-037
NGUT-chr1-071	Ca21chr1	889725	890125	NOVEL-Ca21chr1-038
NGUT-chr1-072	Ca21chr1	991050	991600	ntar96
NGUT-chr1-073	Ca21chr1	1045975	1046575	NOVEL-Ca21chr1-040
NGUT-chr1-074	Ca21chr1	1046675	1047525	NOVEL-Ca21chr1-041
NGUT-chr1-075	Ca21chr1	1074575	1075475	NOVEL-Ca21chr1-042
NGUT-chr1-076	Ca21chr1	1088500	1089250	NOVEL-Ca21chr1-043
NGUT-chr1-077	Ca21chr1	1090600	1091050	NOVEL-Ca21chr1-044
NGUT-chr1-078	Ca21chr1	1096325	1096525	TF1W466
NGUT-chr1-079	Ca21chr1	1096325	1096950	ntar105
NGUT-chr1-080	Ca21chr1	1098950	1099175	ntar106
NGUT-chr1-081	Ca21chr1	1107900	1108200	ntar109
NGUT-chr1-082	Ca21chr1	1157400	1158025	NOVEL-Ca21chr1-045
NGUT-chr1-083	Ca21chr1	1165050	1165575	NOVEL-Ca21chr1-046
NGUT-chr1-084	Ca21chr1	1165900	1166450	NOVEL-Ca21chr1-047/ntar1426
NGUT-chr1-085	Ca21chr1	1191375	1191450	
NGUT-chr1-086	Ca21chr1	1242800	1243750	NOVEL-Ca21chr1-049
NGUT-chr1-087	Ca21chr1	1243975	1244400	NOVEL-Ca21chr1-050
NGUT-chr1-088	Ca21chr1	1249950	1250175	NOVEL-Ca21chr1-051
NGUT-chr1-089	Ca21chr1	1250800	1251200	NOVEL-Ca21chr1-052
NGUT-chr1-090	Ca21chr1	1291650	1292025	NOVEL-Ca21chr1-053
NGUT-chr1-091	Ca21chr1	1303875	1304100	NOVEL-Ca21chr1-054
NGUT-chr1-092	Ca21chr1	1313800	1314225	
NGUT-chr1-093	Ca21chr1	1335150	1335650	NOVEL-Ca21chr1-060
NGUT-chr1-094	Ca21chr1	1411375	1411525	
NGUT-chr1-095	Ca21chr1	1421250	1421600	TF1C404
NGUT-chr1-096	Ca21chr1	1439150	1439600	NOVEL-Ca21chr1-061

NGUT-chr1-097	Ca21chr1	1524525	1525150	
NGUT-chr1-098	Ca21chr1	1535600	1536000	
NGUT-chr1-099	Ca21chr1	1540000	1540600	
NGUT-chr1-100	Ca21chr1	1570900	1571675	NOVEL-Ca21chr1-064
NGUT-chr1-101	Ca21chr1	1578375	1578850	
NGUT-chr1-102	Ca21chr1	1593500	1593875	NOVEL-Ca21chr1-065
NGUT-chr1-103	Ca21chr1	1599275	1600125	NOVEL-Ca21chr1-066
NGUT-chr1-104	Ca21chr1	1614975	1615100	TF1W422
NGUT-chr1-105	Ca21chr1	1630625	1630700	
NGUT-chr1-106	Ca21chr1	1631025	1631100	ntar148
NGUT-chr1-107	Ca21chr1	1631175	1631300	
NGUT-chr1-108	Ca21chr1	1631350	1631425	
NGUT-chr1-109	Ca21chr1	1631550	1631650	
NGUT-chr1-110	Ca21chr1	1636525	1636750	TF1C25
NGUT-chr1-111	Ca21chr1	1657050	1658600	NOVEL-Ca21chr1-067
NGUT-chr1-112	Ca21chr1	1663300	1664075	NOVEL-Ca21chr1-068
NGUT-chr1-113	Ca21chr1	1664475	1665550	NOVEL-Ca21chr1-069
NGUT-chr1-114	Ca21chr1	1665650	1665975	TF1W405
NGUT-chr1-115	Ca21chr1	1673475	1674200	ntar155
NGUT-chr1-116	Ca21chr1	1680650	1681150	NOVEL-Ca21chr1-070
NGUT-chr1-117	Ca21chr1	1681425	1682200	
NGUT-chr1-118	Ca21chr1	1684400	1684900	
NGUT-chr1-119	Ca21chr1	1685000	1685575	NOVEL-Ca21chr1-071
NGUT-chr1-120	Ca21chr1	1685650	1685875	
NGUT-chr1-121	Ca21chr1	1686025	1686725	
NGUT-chr1-122	Ca21chr1	1689200	1690075	
NGUT-chr1-123	Ca21chr1	1714175	1714675	ntar158
NGUT-chr1-124	Ca21chr1	1728250	1728475	TF1W357
NGUT-chr1-125	Ca21chr1	1728650	1729350	ntar161
NGUT-chr1-126	Ca21chr1	1735675	1736100	NOVEL-Ca21chr1-074
NGUT-chr1-127	Ca21chr1	1739000	1739525	NOVEL-Ca21chr1-075
NGUT-chr1-128	Ca21chr1	1743550	1744075	NOVEL-Ca21chr1-078
NGUT-chr1-129	Ca21chr1	1784400	1784900	NOVEL-Ca21chr1-077
NGUT-chr1-130	Ca21chr1	1784925	1785250	
NGUT-chr1-131	Ca21chr1	1800825	1801375	ntar171
NGUT-chr1-132	Ca21chr1	1825850	1826550	NOVEL-Ca21chr1-080
NGUT-chr1-133	Ca21chr1	1833350	1833900	NOVEL-Ca21chr1-081
NGUT-chr1-134	Ca21chr1	1943000	1943225	TF1C230
NGUT-chr1-135	Ca21chr1	1943400	1943650	TF1W231
NGUT-chr1-136	Ca21chr1	1944275	1944825	
NGUT-chr1-137	Ca21chr1	1945825	1946150	NOVEL-Ca21chr1-081
NGUT-chr1-138	Ca21chr1	1947300	1947800	NOVEL-Ca21chr1-082
NGUT-chr1-139	Ca21chr1	1952475	1953225	TF1W393
NGUT-chr1-140	Ca21chr1	1955750	1956075	NOVEL-Ca21chr1-085
NGUT-chr1-141	Ca21chr1	1984850	1985150	
NGUT-chr1-142	Ca21chr1	1995300	1996025	NOVEL-Ca21chr1-87
NGUT-chr1-143	Ca21chr1	2024625	2025400	NOVEL-Ca21chr1-89
NGUT-chr1-144	Ca21chr1	2053200	2054050	NOVEL-Ca21chr1-90
NGUT-chr1-145	Ca21chr1	2100775	2102100	NOVEL-Ca21chr1-91
NGUT-chr1-146	Ca21chr1	2105325	2105900	NOVEL-Ca21chr1-92

NGUT-chr1-147	Ca21chr1	2106025	2106800	NOVEL-Ca21chr1-93
NGUT-chr1-148	Ca21chr1	2116300	2117400	NOVEL-Ca21chr1-94/95
NGUT-chr1-149	Ca21chr1	2164300	2165300	NOVEL-Ca21chr1-96
NGUT-chr1-150	Ca21chr1	2169450	2169700	
NGUT-chr1-151	Ca21chr1	2185450	2185900	NOVEL-Ca21chr1-97
NGUT-chr1-152	Ca21chr1	2194900	2195100	TF1W419
NGUT-chr1-153	Ca21chr1	2213025	2213300	
NGUT-chr1-154	Ca21chr1	2217600	2217900	TF1W455
NGUT-chr1-155	Ca21chr1	2242825	2243175	TF1C247/TF1W248
NGUT-chr1-156	Ca21chr1	2243250	2244400	
NGUT-chr1-157	Ca21chr1	2244725	2244975	
NGUT-chr1-158	Ca21chr1	2279075	2279375	TF1C13
NGUT-chr1-159	Ca21chr1	2281400	2281775	TF1W334
NGUT-chr1-160	Ca21chr1	2290000	2290600	NOVEL-Ca21chr1-99
NGUT-chr1-161	Ca21chr1	2295250	2295850	NOVEL-Ca21chr1-100
NGUT-chr1-162	Ca21chr1	2303000	2303300	NOVEL-Ca21chr1-101
NGUT-chr1-163	Ca21chr1	2345400	2345800	NOVEL-Ca21chr1-102
NGUT-chr1-164	Ca21chr1	2346700	2347125	NOVEL-Ca21chr1-103
NGUT-chr1-165	Ca21chr1	2351900	2352225	TF1C14
NGUT-chr1-166	Ca21chr1	2401925	2402525	
NGUT-chr1-167	Ca21chr1	2474600	2474875	NOVEL-Ca21chr1-105
NGUT-chr1-168	Ca21chr1	2483900	2484100	
NGUT-chr1-169	Ca21chr1	2484300	2484575	
NGUT-chr1-170	Ca21chr1	2486225	2486800	NOVEL-Ca21chr1-106
NGUT-chr1-171	Ca21chr1	2512500	2513125	NOVEL-Ca21chr1-107
NGUT-chr1-172	Ca21chr1	2515725	2516100	NOVEL-Ca21chr1-108
NGUT-chr1-173	Ca21chr1	2516625	2517050	NOVEL-Ca21chr1-109
NGUT-chr1-174	Ca21chr1	2583650	2584000	TF1W431
NGUT-chr1-175	Ca21chr1	2592325	2593100	NOVEL-Ca21chr1-111
NGUT-chr1-176	Ca21chr1	2631575	2631975	NOVEL-Ca21chr1-112
NGUT-chr1-177	Ca21chr1	2694975	2695125	TF1W80
NGUT-chr1-178	Ca21chr1	2719225	2719500	NOVEL-Ca21chr1-114
NGUT-chr1-179	Ca21chr1	2722300	2723225	NOVEL-Ca21chr1-115
NGUT-chr1-180	Ca21chr1	2751025	2751450	
NGUT-chr1-181	Ca21chr1	2788350	2789000	NOVEL-Ca21chr1-116
NGUT-chr1-182	Ca21chr1	2796750	2797350	
NGUT-chr1-183	Ca21chr1	2799150	2799575	TF1C282/TF1W383
NGUT-chr1-184	Ca21chr1	2800500	2800725	TF1C385
NGUT-chr1-185	Ca21chr1	2806975	2807325	TF1W15
NGUT-chr1-186	Ca21chr1	2810325	2810975	NOVEL-Ca21chr1-118
NGUT-chr1-187	Ca21chr1	2830775	2831700	NOVEL-Ca21chr1-121
NGUT-chr1-188	Ca21chr1	2839150	2839575	NOVEL-Ca21chr1-122
NGUT-chr1-189	Ca21chr1	2872025	2872600	NOVEL-Ca21chr1-124
NGUT-chr1-190	Ca21chr1	2872900	2873375	NOVEL-Ca21chr1-125
NGUT-chr1-191	Ca21chr1	2883475	2883650	
NGUT-chr1-192	Ca21chr1	2901750	2902275	TF1W289
NGUT-chr1-193	Ca21chr1	2908825	2909675	NOVEL-Ca21chr1-126
NGUT-chr1-194	Ca21chr1	2924175	2924575	NOVEL-Ca21chr1-127
NGUT-chr1-195	Ca21chr1	2954450	2954850	TF1W295
NGUT-chr1-196	Ca21chr1	2972925	2973525	NOVEL-Ca21chr1-128

NGUT-chr1-197	Ca21chr1	2979550	2979825	
NGUT-chr1-198	Ca21chr1	2990575	2991275	
NGUT-chr1-199	Ca21chr1	2999250	2999575	NOVEL-Ca21chr1-129
NGUT-chr1-200	Ca21chr1	3025500	3025800	
NGUT-chr1-201	Ca21chr1	3049000	3049775	NOVEL-Ca21chr1-133
NGUT-chr1-202	Ca21chr1	3057325	3058000	TF1C390
NGUT-chr1-203	Ca21chr1	3110850	3111800	
NGUT-chr1-204	Ca21chr1	3119350	3119800	
NGUT-chr1-205	Ca21chr1	3120525	3120875	TF1C460
NGUT-chr1-206	Ca21chr1	3132075	3132800	NOVEL-Ca21chr1-135
NGUT-chr1-207	Ca21chr1	3161300	3161925	TF1W308
NGUT-chr2-001	Ca21chr2	0	375	
NGUT-chr2-002	Ca21chr2	575	775	NOVEL-Ca21chr2-001
NGUT-chr2-003	Ca21chr2	3550	3850	
NGUT-chr2-004	Ca21chr2	17725	18625	NOVEL-Ca21chr2-003
NGUT-chr2-005	Ca21chr2	129150	129400	TF2W92
NGUT-chr2-006	Ca21chr2	147800	148000	
NGUT-chr2-007	Ca21chr2	161900	162925	NOVEL-Ca21chr2-004
NGUT-chr2-008	Ca21chr2	163700	164075	NOVEL-Ca21chr2-005
NGUT-chr2-009	Ca21chr2	177100	177750	NOVEL-Ca21chr2-006
NGUT-chr2-010	Ca21chr2	199150	200425	NOVEL-Ca21chr2-008
NGUT-chr2-011	Ca21chr2	204125	204625	NOVEL-Ca21chr2-010
NGUT-chr2-012	Ca21chr2	217400	218375	NOVEL-Ca21chr2-011
NGUT-chr2-013	Ca21chr2	218450	219025	NOVEL-Ca21chr2-012
NGUT-chr2-014	Ca21chr2	222300	223200	NOVEL-Ca21chr2-013
NGUT-chr2-015	Ca21chr2	250825	251550	NOVEL-Ca21chr2-014
NGUT-chr2-016	Ca21chr2	289450	289650	TF2W344
NGUT-chr2-017	Ca21chr2	316800	317250	
NGUT-chr2-018	Ca21chr2	368675	368950	NOVEL-Ca21chr2-017
NGUT-chr2-019	Ca21chr2	369300	369825	
NGUT-chr2-020	Ca21chr2	407375	407800	NOVEL-Ca21chr2-018
NGUT-chr2-021	Ca21chr2	442750	443375	
NGUT-chr2-022	Ca21chr2	559600	559900	NOVEL-Ca21chr2-020
NGUT-chr2-023	Ca21chr2	581150	581500	
NGUT-chr2-024	Ca21chr2	582025	582725	NOVEL-Ca21chr2-021
NGUT-chr2-025	Ca21chr2	608475	606825	TF2C274
NGUT-chr2-026	Ca21chr2	609625	610400	NOVEL-Ca21chr2-023
NGUT-chr2-027	Ca21chr2	626550	627950	
NGUT-chr2-028	Ca21chr2	642850	643300	
NGUT-chr2-029	Ca21chr2	658450	658725	NOVEL-Ca21chr2-024
NGUT-chr2-030	Ca21chr2	660525	661950	
NGUT-chr2-031	Ca21chr2	663075	663475	TF2W19
NGUT-chr2-032	Ca21chr2	670075	671475	NOVEL-Ca21chr2-025
NGUT-chr2-033	Ca21chr2	671375	671925	
NGUT-chr2-034	Ca21chr2	731675	733450	NOVEL-Ca21chr2-026
NGUT-chr2-035	Ca21chr2	733700	734700	NOVEL-Ca21chr2-027
NGUT-chr2-036	Ca21chr2	737650	738650	
NGUT-chr2-037	Ca21chr2	739200	739900	TF2W141/TF2C142
NGUT-chr2-038	Ca21chr2	742650	743650	
NGUT-chr2-039	Ca21chr2	757700	758025	TF2W348

NGUT-chr2-040	Ca21chr2	863900	864050	TF2C53
NGUT-chr2-041	Ca21chr2	869750	870175	TF2W150
NGUT-chr2-042	Ca21chr2	870425	870625	TF2W151
NGUT-chr2-043	Ca21chr2	920225	920500	
NGUT-chr2-044	Ca21chr2	939800	940200	NOVEL-Ca21chr2-028
NGUT-chr2-045	Ca21chr2	951700	952250	ntar372
NGUT-chr2-046	Ca21chr2	962525	962850	NOVEL-Ca21chr2-029
NGUT-chr2-047	Ca21chr2	963050	964525	NOVEL-Ca21chr2-030
NGUT-chr2-048	Ca21chr2	968575	968850	
NGUT-chr2-049	Ca21chr2	968900	969225	NOVEL-Ca21chr2-031
NGUT-chr2-050	Ca21chr2	969500	970325	NOVEL-Ca21chr2-032
NGUT-chr2-051	Ca21chr2	970600	971100	NOVEL-Ca21chr2-033
NGUT-chr2-052	Ca21chr2	971200	971700	ntar379
NGUT-chr2-053	Ca21chr2	990350	990950	NOVEL-Ca21chr2-037
NGUT-chr2-054	Ca21chr2	1022375	1022875	NOVEL-Ca21chr2-038
NGUT-chr2-055	Ca21chr2	1024325	1025325	NOVEL-Ca21chr2-039
NGUT-chr2-056	Ca21chr2	1025450	1025850	NOVEL-Ca21chr2-040
NGUT-chr2-057	Ca21chr2	1026500	1026975	NOVEL-Ca21chr2-041
NGUT-chr2-058	Ca21chr2	1032250	1032425	NOVEL-Ca21chr2-042
NGUT-chr2-059	Ca21chr2	1035625	1035875	NOVEL-Ca21chr2-043
NGUT-chr2-060	Ca21chr2	1080000	1080325	ntar390
NGUT-chr2-061	Ca21chr2	1082475	1082825	ntar391
NGUT-chr2-062	Ca21chr2	1104750	1104900	
NGUT-chr2-063	Ca21chr2	1111700	1112125	NOVEL-Ca21chr2-044
NGUT-chr2-064	Ca21chr2	1117750	1118300	NOVEL-Ca21chr2-045
NGUT-chr2-065	Ca21chr2	1134275	1134650	
NGUT-chr2-066	Ca21chr2	1141125	1141400	TF2W340
NGUT-chr2-067	Ca21chr2	1142525	1142850	TF2C341
NGUT-chr2-068	Ca21chr2	1147000	1148400	NOVEL-Ca21chr2-046
NGUT-chr2-069	Ca21chr2	1156000	1156600	NOVEL-Ca21chr2-047
NGUT-chr2-070	Ca21chr2	1232000	1232425	NOVEL-Ca21chr2-048
NGUT-chr2-071	Ca21chr2	1304550	1304900	NOVEL-Ca21chr2-049
NGUT-chr2-072	Ca21chr2	1332450	1333450	NOVEL-Ca21chr2-050
NGUT-chr2-073	Ca21chr2	1344500	1344825	TF2C12
NGUT-chr2-074	Ca21chr2	1345300	1345700	
NGUT-chr2-075	Ca21chr2	1348700	1348950	TF2C255
NGUT-chr2-076	Ca21chr2	1362200	1362500	NOVEL-Ca21chr2-051
NGUT-chr2-077	Ca21chr2	1363000	1364025	NOVEL-Ca21chr2-052
NGUT-chr2-078	Ca21chr2	1368950	1369475	TF2W23
NGUT-chr2-079	Ca21chr2	1369900	1370200	TF2W24
NGUT-chr2-080	Ca21chr2	1377850	1378625	NOVEL-Ca21chr2-054
NGUT-chr2-081	Ca21chr2	1382075	1382425	
NGUT-chr2-082	Ca21chr2	1421625	1422125	NOVEL-Ca21chr2-056
NGUT-chr2-083	Ca21chr2	1425800	1426250	NOVEL-Ca21chr2-058
NGUT-chr2-084	Ca21chr2	1452100	1452375	
NGUT-chr2-085	Ca21chr2	1455825	1456125	TF2C191
NGUT-chr2-086	Ca21chr2	1456550	1457275	NOVEL-Ca21chr2-059
NGUT-chr2-087	Ca21chr2	1465575	1466200	NOVEL-Ca21chr2-060
NGUT-chr2-088	Ca21chr2	1497875	1498400	NOVEL-Ca21chr2-061
NGUT-chr2-089	Ca21chr2	1544900	1545400	TF2C5

NGUT-chr2-090	Ca21chr2	1587000	1587200	
NGUT-chr2-091	Ca21chr2	1605825	1606300	
NGUT-chr2-092	Ca21chr2	1606650	1607625	NOVEL-Ca21chr2-062
NGUT-chr2-093	Ca21chr2	1637875	1638250	TF2W333
NGUT-chr2-094	Ca21chr2	1656150	1656525	
NGUT-chr2-095	Ca21chr2	1674800	1675400	NOVEL-Ca21chr2-063
NGUT-chr2-096	Ca21chr2	1697425	1698325	NOVEL-Ca21chr2-065
NGUT-chr2-097	Ca21chr2	1703800	1704400	NOVEL-Ca21chr2-066
NGUT-chr2-098	Ca21chr2	1714375	1714875	NOVEL-Ca21chr2-067
NGUT-chr2-099	Ca21chr2	1718750	1719225	NOVEL-Ca21chr2-068
NGUT-chr2-100	Ca21chr2	1723925	1724225	TF2C9
NGUT-chr2-101	Ca21chr2	1741200	1742325	NOVEL-Ca21chr2-069
NGUT-chr2-102	Ca21chr2	1742575	1743050	NOVEL-Ca21chr2-070
NGUT-chr2-103	Ca21chr2	1743325	1744175	NOVEL-Ca21chr2-071
NGUT-chr2-104	Ca21chr2	1744300	1746375	NOVEL-Ca21chr2-072
NGUT-chr2-105	Ca21chr2	1797050	1797400	TF2W77
NGUT-chr2-106	Ca21chr2	1802725	1803575	TF2C213
NGUT-chr2-107	Ca21chr2	1837100	1838050	NOVEL-Ca21chr2-073
NGUT-chr2-108	Ca21chr2	1838400	1839250	TF2C311
NGUT-chr2-109	Ca21chr2	1851200	1851550	TF2W25
NGUT-chr2-110	Ca21chr2	1864925	1865300	NOVEL-Ca21chr2-075
NGUT-chr2-111	Ca21chr2	1878600	1878875	NOVEL-Ca21chr2-077
NGUT-chr2-112	Ca21chr2	1879400	1879675	
NGUT-chr2-113	Ca21chr2	1886150	1886500	TF2C318
NGUT-chr2-114	Ca21chr2	1886700	1886850	
NGUT-chr2-115	Ca21chr2	1887375	1887825	NOVEL-Ca21chr2-078
NGUT-chr2-116	Ca21chr2	1914950	1915400	NOVEL-Ca21chr2-079
NGUT-chr2-117	Ca21chr2	1918800	1919125	TF2W268
NGUT-chr2-118	Ca21chr2	1923250	1925400	NOVEL-Ca21chr2-080
NGUT-chr2-119	Ca21chr2	1929775	1930100	
NGUT-chr2-120	Ca21chr2	1935525	1936200	
NGUT-chr2-121	Ca21chr2	1948150	1948400	
NGUT-chr2-122	Ca21chr2	1953775	1954350	NOVEL-Ca21chr2-081
NGUT-chr2-123	Ca21chr2	1959250	1960050	NOVEL-Ca21chr2-082
NGUT-chr2-124	Ca21chr2	1963325	1964000	
NGUT-chr2-125	Ca21chr2	1976950	1977325	NOVEL-Ca21chr2-083
NGUT-chr2-126	Ca21chr2	2008800	2009150	
NGUT-chr2-127	Ca21chr2	2032475	2032975	NOVEL-Ca21chr2-084
NGUT-chr2-128	Ca21chr2	2033075	2034375	NOVEL-Ca21chr2-085
NGUT-chr2-129	Ca21chr2	2039850	2041275	NOVEL-Ca21chr2-086
NGUT-chr2-130	Ca21chr2	2047650	2047975	NOVEL-Ca21chr2-087
NGUT-chr2-131	Ca21chr2	2063600	2064400	NOVEL-Ca21chr2-088
NGUT-chr2-132	Ca21chr2	2066350	2066650	NOVEL-Ca21chr2-089
NGUT-chr2-133	Ca21chr2	2073950	2074500	NOVEL-Ca21chr2-090
NGUT-chr2-134	Ca21chr2	2080575	2080875	TF2C235
NGUT-chr2-135	Ca21chr2	2086425	2087100	TF2C258
NGUT-chr2-136	Ca21chr2	2097975	2098300	NOVEL-Ca21chr2-091
NGUT-chr2-137	Ca21chr2	2106100	2107100	NOVEL-Ca21chr2-092
NGUT-chr2-138	Ca21chr2	2114075	2114350	TF2C83
NGUT-chr2-139	Ca21chr2	2119700	2120225	NOVEL-Ca21chr2-093

NGUT-chr2-140	Ca21chr2	2134625	2135125	
NGUT-chr2-141	Ca21chr2	2139900	2140075	
NGUT-chr2-142	Ca21chr2	2140100	2140475	NOVEL-Ca21chr2-095
NGUT-chr2-143	Ca21chr2	2140525	2140725	TF2W84
NGUT-chr2-144	Ca21chr2	2141300	2141675	NOVEL-Ca21chr2-096
NGUT-chr2-145	Ca21chr2	2156250	2156575	TF2C26
NGUT-chr2-146	Ca21chr2	2179525	2179700	TF2W338
NGUT-chr2-147	Ca21chr2	2187700	2188050	
NGUT-chr2-148	Ca21chr2	2231050	2231200	
NGUT-chr2-149	Ca21chr2	2231750	2231825	
NGUT-chr3-001	Ca21chr3	1550	2025	NOVEL-Ca21chr3-001
NGUT-chr3-002	Ca21chr3	2200	3325	NOVEL-Ca21chr3-002
NGUT-chr3-003	Ca21chr3	8325	8425	
NGUT-chr3-004	Ca21chr3	10550	11150	
NGUT-chr3-005	Ca21chr3	12325	12725	
NGUT-chr3-006	Ca21chr3	15350	15600	
NGUT-chr3-007	Ca21chr3	50000	50575	
NGUT-chr3-008	Ca21chr3	80575	80725	
NGUT-chr3-009	Ca21chr3	97375	97525	TF3C20
NGUT-chr3-010	Ca21chr3	127050	127475	NOVEL-Ca21chr3-003
NGUT-chr3-011	Ca21chr3	127600	127875	NOVEL-Ca21chr3-004
NGUT-chr3-012	Ca21chr3	130350	130725	
NGUT-chr3-013	Ca21chr3	136300	136600	
NGUT-chr3-014	Ca21chr3	137200	137425	
NGUT-chr3-015	Ca21chr3	152250	152925	
NGUT-chr3-016	Ca21chr3	153600	154300	NOVEL-Ca21chr3-005
NGUT-chr3-017	Ca21chr3	216225	216400	TF3W71
NGUT-chr3-018	Ca21chr3	234925	235075	TF3C75
NGUT-chr3-019	Ca21chr3	235175	235775	NOVEL-Ca21chr3-006
NGUT-chr3-020	Ca21chr3	245075	245325	NOVEL-Ca21chr3-007
NGUT-chr3-021	Ca21chr3	247700	247950	
NGUT-chr3-022	Ca21chr3	258900	259350	TF3W21
NGUT-chr3-023	Ca21chr3	263825	264375	NOVEL-Ca21chr3-008
NGUT-chr3-024	Ca21chr3	296400	296550	TF3C82
NGUT-chr3-025	Ca21chr3	304025	304650	NOVEL-Ca21chr3-010
NGUT-chr3-026	Ca21chr3	313800	314425	NOVEL-Ca21chr3-011
NGUT-chr3-027	Ca21chr3	314575	314900	TF3C38
NGUT-chr3-028	Ca21chr3	333450	334425	NOVEL-Ca21chr3-012
NGUT-chr3-029	Ca21chr3	361825	362425	NOVEL-Ca21chr3-013
NGUT-chr3-030	Ca21chr3	364250	365100	NOVEL-Ca21chr3-014
NGUT-chr3-031	Ca21chr3	366700	366875	
NGUT-chr3-032	Ca21chr3	397150	397300	
NGUT-chr3-033	Ca21chr3	397925	398275	
NGUT-chr3-034	Ca21chr3	401050	401325	
NGUT-chr3-035	Ca21chr3	434675	435600	NOVEL-Ca21chr3-017
NGUT-chr3-036	Ca21chr3	458675	458950	
NGUT-chr3-037	Ca21chr3	486600	486775	
NGUT-chr3-038	Ca21chr3	491025	491950	NOVEL-Ca21chr3-018
NGUT-chr3-039	Ca21chr3	493100	493300	
NGUT-chr3-040	Ca21chr3	521250	521550	NOVEL-Ca21chr3-020

NGUT-chr3-041	Ca21chr3	523000	523300	TF3C96
NGUT-chr3-042	Ca21chr3	566475	567200	
NGUT-chr3-043	Ca21chr3	568275	568600	TF3W11
NGUT-chr3-044	Ca21chr3	573250	573925	NOVEL-Ca21chr3-021
NGUT-chr3-045	Ca21chr3	683650	684425	NOVEL-Ca21chr3-022
NGUT-chr3-046	Ca21chr3	703200	703900	NOVEL-Ca21chr3-023
NGUT-chr3-047	Ca21chr3	706600	707000	NOVEL-Ca21chr3-024
NGUT-chr3-048	Ca21chr3	707225	707425	
NGUT-chr3-049	Ca21chr3	790100	790725	NOVEL-Ca21chr3-025
NGUT-chr3-050	Ca21chr3	790925	791200	
NGUT-chr3-051	Ca21chr3	821775	822325	
NGUT-chr3-052	Ca21chr3	847650	848125	
NGUT-chr3-053	Ca21chr3	972300	973350	NOVEL-Ca21chr3-028
NGUT-chr3-054	Ca21chr3	984200	984500	
NGUT-chr3-055	Ca21chr3	1040425	1041175	TF3W230
NGUT-chr3-056	Ca21chr3	1046800	1047675	
NGUT-chr3-057	Ca21chr3	1057700	1058200	
NGUT-chr3-058	Ca21chr3	1058375	1059125	NOVEL-Ca21chr3-029
NGUT-chr3-059	Ca21chr3	1088850	1089850	TF3C24/TF3C13
NGUT-chr3-060	Ca21chr3	1109300	1109900	
NGUT-chr3-061	Ca21chr3	1110075	1111150	
NGUT-chr3-062	Ca21chr3	1121850	1122150	
NGUT-chr3-063	Ca21chr3	1123400	1123675	NOVEL-Ca21chr3-030
NGUT-chr3-064	Ca21chr3	1129600	1129975	NOVEL-Ca21chr3-031
NGUT-chr3-065	Ca21chr3	1156650	1157325	NOVEL-Ca21chr3-032
NGUT-chr3-066	Ca21chr3	1157525	1157825	
NGUT-chr3-067	Ca21chr3	1162875	1163600	
NGUT-chr3-068	Ca21chr3	1167900	1168200	TF3W25
NGUT-chr3-069	Ca21chr3	1175475	1176000	
NGUT-chr3-070	Ca21chr3	1177400	1177825	NOVEL-Ca21chr3-033
NGUT-chr3-071	Ca21chr3	1191925	1192625	NOVEL-Ca21chr3-034
NGUT-chr3-072	Ca21chr3	1194700	1195000	NOVEL-Ca21chr3-035
NGUT-chr3-073	Ca21chr3	1201250	1201725	
NGUT-chr3-074	Ca21chr3	1215400	1215875	
NGUT-chr3-075	Ca21chr3	1218525	1219400	NOVEL-Ca21chr3-039
NGUT-chr3-076	Ca21chr3	1220325	1220725	NOVEL-Ca21chr3-040
NGUT-chr3-077	Ca21chr3	1221225	1221600	
NGUT-chr3-078	Ca21chr3	1221700	1222025	
NGUT-chr3-079	Ca21chr3	1222100	1222725	
NGUT-chr3-080	Ca21chr3	1224000	1224600	NOVEL-Ca21chr3-041
NGUT-chr3-081	Ca21chr3	1239425	1239800	NOVEL-Ca21chr3-042
NGUT-chr3-082	Ca21chr3	1262250	1263200	NOVEL-Ca21chr3-043
NGUT-chr3-083	Ca21chr3	1268100	1268400	NOVEL-Ca21chr3-044
NGUT-chr3-084	Ca21chr3	1268500	1268575	TF3W143
NGUT-chr3-085	Ca21chr3	1268650	1269650	NOVEL-Ca21chr3-045
NGUT-chr3-086	Ca21chr3	1271650	1272000	TF3W52
NGUT-chr3-087	Ca21chr3	1273200	1274525	NOVEL-Ca21chr3-046
NGUT-chr3-088	Ca21chr3	1277825	1278800	NOVEL-Ca21chr3-047
NGUT-chr3-089	Ca21chr3	1285350	1285675	NOVEL-Ca21chr3-048
NGUT-chr3-090	Ca21chr3	1289150	1289325	TF3C150

NGUT-chr3-091	Ca21chr3	1303100	1303175	
NGUT-chr3-092	Ca21chr3	1304800	1305250	NOVEL-Ca21chr3-049
NGUT-chr3-093	Ca21chr3	1320650	1321125	NOVEL-Ca21chr3-050
NGUT-chr3-094	Ca21chr3	1321225	1321500	NOVEL-Ca21chr3-051
NGUT-chr3-095	Ca21chr3	1332925	1333625	
NGUT-chr3-096	Ca21chr3	1360325	1361250	NOVEL-Ca21chr3-052
NGUT-chr3-097	Ca21chr3	1361375	1361675	NOVEL-Ca21chr3-053
NGUT-chr3-098	Ca21chr3	1387100	1387650	
NGUT-chr3-099	Ca21chr3	1393525	1393725	TF3W58
NGUT-chr3-100	Ca21chr3	1396100	1396750	NOVEL-Ca21chr3-054
NGUT-chr3-101	Ca21chr3	1407225	1407675	NOVEL-Ca21chr3-055
NGUT-chr3-102	Ca21chr3	1470175	1470350	
NGUT-chr3-103	Ca21chr3	1470500	1470700	
NGUT-chr3-104	Ca21chr3	1473825	1473975	
NGUT-chr3-105	Ca21chr3	1475925	1476900	NOVEL-Ca21chr3-056
NGUT-chr3-106	Ca21chr3	1480100	1480425	NOVEL-Ca21chr3-057
NGUT-chr3-107	Ca21chr3	1490475	1490900	TF3C207
NGUT-chr3-108	Ca21chr3	1495150	1496000	
NGUT-chr3-109	Ca21chr3	1531025	1531475	NOVEL-Ca21chr3-059
NGUT-chr3-110	Ca21chr3	1548750	1548900	
NGUT-chr3-111	Ca21chr3	1567800	1568450	ntar663
NGUT-chr3-112	Ca21chr3	1574275	1574750	TF3C214
NGUT-chr3-113	Ca21chr3	1588375	1588700	
NGUT-chr3-114	Ca21chr3	1589400	1589625	
NGUT-chr3-115	Ca21chr3	1589750	1590200	ntar667
NGUT-chr3-116	Ca21chr3	1605925	1607400	NOVEL-Ca21chr3-060
NGUT-chr3-117	Ca21chr3	1607525	1607900	TF3W186
NGUT-chr3-118	Ca21chr3	1619175	1619325	TF3C174/TF3W60
NGUT-chr3-119	Ca21chr3	1637475	1637750	ntar673
NGUT-chr3-120	Ca21chr3	1642925	1643600	ntar675
NGUT-chr3-121	Ca21chr3	1646050	1646600	
NGUT-chr3-122	Ca21chr3	1647500	1648425	NOVEL-Ca21chr3-061
NGUT-chr3-123	Ca21chr3	1648725	1649500	NOVEL-Ca21chr3-062
NGUT-chr3-124	Ca21chr3	1651750	1652350	
NGUT-chr3-125	Ca21chr3	1672100	1672525	
NGUT-chr3-126	Ca21chr3	1722200	1723275	
NGUT-chr3-127	Ca21chr3	1724025	1724500	NOVEL-Ca21chr3-064
NGUT-chr3-128	Ca21chr3	1761425	1761725	TF3W223
NGUT-chr3-129	Ca21chr3	1786675	1786825	TF3W27
NGUT-chr3-130	Ca21chr3	1787275	1787500	ntar691
NGUT-chr3-131	Ca21chr3	1790225	1790425	
NGUT-chr3-132	Ca21chr3	1793600	1794025	NOVEL-Ca21chr3-065
NGUT-chr3-133	Ca21chr3	1794150	1795000	ntar693/694
NGUT-chr3-134	Ca21chr3	1795475	1795925	
NGUT-chr3-135	Ca21chr3	1796425	1796525	
NGUT-chr3-136	Ca21chr3	1796750	1797150	NOVEL-Ca21chr3-067
NGUT-chr3-137	Ca21chr3	1797600	1797775	TF3W4
NGUT-chr3-138	Ca21chr3	1798000	1798250	ntar697
NGUT-chr4-001	Ca21chr4	2150	2700	NOVEL-Ca21chr4-001
NGUT-chr4-002	Ca21chr4	55550	56325	NOVEL-Ca21chr4-002

NGUT-chr4-003	Ca21chr4	67100	67650	
NGUT-chr4-004	Ca21chr4	71975	72150	
NGUT-chr4-005	Ca21chr4	80400	81250	NOVEL-Ca21chr4-003
NGUT-chr4-006	Ca21chr4	83700	83900	
NGUT-chr4-007	Ca21chr4	97175	97400	ntar709
NGUT-chr4-008	Ca21chr4	127425	128025	NOVEL-Ca21chr4-004
NGUT-chr4-009	Ca21chr4	128350	128775	
NGUT-chr4-010	Ca21chr4	129700	130225	ntar714
NGUT-chr4-011	Ca21chr4	130300	130775	ntar715
NGUT-chr4-012	Ca21chr4	131050	131275	TF4C194
NGUT-chr4-013	Ca21chr4	147500	147950	ntar717
NGUT-chr4-014	Ca21chr4	164400	164650	NOVEL-Ca21chr4-006
NGUT-chr4-015	Ca21chr4	172725	173025	NOVEL-Ca21chr4-007
NGUT-chr4-016	Ca21chr4	173300	173550	NOVEL-Ca21chr4-007
NGUT-chr4-017	Ca21chr4	191575	191850	ntar724
NGUT-chr4-018	Ca21chr4	194575	194825	TF4C19
NGUT-chr4-019	Ca21chr4	201650	201950	ntar726
NGUT-chr4-020	Ca21chr4	237975	238800	
NGUT-chr4-021	Ca21chr4	239200	239725	
NGUT-chr4-022	Ca21chr4	252300	252775	ntar728
NGUT-chr4-023	Ca21chr4	262100	262500	NOVEL-Ca21chr4-008
NGUT-chr4-024	Ca21chr4	276200	276575	NOVEL-Ca21chr4-011
NGUT-chr4-025	Ca21chr4	277075	277675	NOVEL-Ca21chr4-012
NGUT-chr4-026	Ca21chr4	279175	279700	
NGUT-chr4-027	Ca21chr4	279150	280150	NOVEL-Ca21chr4-013
NGUT-chr4-028	Ca21chr4	281875	282750	NOVEL-Ca21chr4-014
NGUT-chr4-029	Ca21chr4	290875	291350	ntar740
NGUT-chr4-030	Ca21chr4	316375	316875	TF4C72
NGUT-chr4-031	Ca21chr4	319775	320025	TF4W16
NGUT-chr4-032	Ca21chr4	324950	325625	NOVEL-Ca21chr4-017
NGUT-chr4-033	Ca21chr4	325925	327500	NOVEL-Ca21chr4-018
NGUT-chr4-034	Ca21chr4	337750	338700	NOVEL-Ca21chr4-019
NGUT-chr4-035	Ca21chr4	339225	339675	ntar745
NGUT-chr4-036	Ca21chr4	347750	347900	TF4C64
NGUT-chr4-037	Ca21chr4	374025	374200	TF4C23
NGUT-chr4-038	Ca21chr4	375550	375975	NOVEL-Ca21chr4-020
NGUT-chr4-039	Ca21chr4	381875	382050	
NGUT-chr4-040	Ca21chr4	393225	393675	
NGUT-chr4-041	Ca21chr4	404300	404500	
NGUT-chr4-042	Ca21chr4	470125	470575	NOVEL-Ca21chr4-022
NGUT-chr4-043	Ca21chr4	473500	473900	TF4C71
NGUT-chr4-044	Ca21chr4	474800	476400	NOVEL-Ca21chr4-023
NGUT-chr4-045	Ca21chr4	476525	476875	NOVEL-Ca21chr4-024
NGUT-chr4-046	Ca21chr4	476925	477050	TF4W25
NGUT-chr4-047	Ca21chr4	477450	478200	NOVEL-Ca21chr4-025/26
NGUT-chr4-048	Ca21chr4	478500	478800	
NGUT-chr4-049	Ca21chr4	479025	479125	TF4C73
NGUT-chr4-050	Ca21chr4	481950	482450	NOVEL-Ca21chr4-027
NGUT-chr4-051	Ca21chr4	520400	520875	TF4C11
NGUT-chr4-052	Ca21chr4	520900	521250	NOVEL-Ca21chr4-029

NGUT-chr4-053	Ca21chr4	521325	522550	NOVEL-Ca21chr4-029
NGUT-chr4-054	Ca21chr4	523000	523575	ntar766
NGUT-chr4-055	Ca21chr4	546275	546800	ntar768
NGUT-chr4-056	Ca21chr4	560850	561150	NOVEL-Ca21chr4-031
NGUT-chr4-057	Ca21chr4	562425	562800	NOVEL-Ca21chr4-032
NGUT-chr4-058	Ca21chr4	574075	574300	NOVEL-Ca21chr4-033
NGUT-chr4-059	Ca21chr4	574525	575100	ntar770
NGUT-chr4-060	Ca21chr4	576425	576825	NOVEL-Ca21chr4-035
NGUT-chr4-061	Ca21chr4	636000	636650	NOVEL-Ca21chr4-036
NGUT-chr4-062	Ca21chr4	637650	638050	ntar775
NGUT-chr4-063	Ca21chr4	648575	648950	ntar776
NGUT-chr4-064	Ca21chr4	667225	667950	
NGUT-chr4-065	Ca21chr4	670725	670900	ntar777
NGUT-chr4-066	Ca21chr4	770025	770375	NOVEL-Ca21chr4-037
NGUT-chr4-067	Ca21chr4	774700	779050	NOVEL-Ca21chr4-038
NGUT-chr4-068	Ca21chr4	780500	780825	NOVEL-Ca21chr4-039
NGUT-chr4-069	Ca21chr4	781650	781975	NOVEL-Ca21chr4-040
NGUT-chr4-070	Ca21chr4	824175	824750	NOVEL-Ca21chr4-041
NGUT-chr4-071	Ca21chr4	830750	831600	NOVEL-Ca21chr4-042
NGUT-chr4-072	Ca21chr4	842250	843100	NOVEL-Ca21chr4-043
NGUT-chr4-073	Ca21chr4	843300	844200	NOVEL-Ca21chr4-044
NGUT-chr4-074	Ca21chr4	851300	851475	
NGUT-chr4-075	Ca21chr4	851950	852100	ntar790
NGUT-chr4-076	Ca21chr4	886925	887400	NOVEL-Ca21chr4-045
NGUT-chr4-077	Ca21chr4	887800	888075	ntar794
NGUT-chr4-078	Ca21chr4	894000	894325	NOVEL-Ca21chr4-046
NGUT-chr4-079	Ca21chr4	935900	936125	
NGUT-chr4-080	Ca21chr4	940825	941200	NOVEL-Ca21chr4-047
NGUT-chr4-081	Ca21chr4	942025	942600	
NGUT-chr4-082	Ca21chr4	959325	960350	NOVEL-Ca21chr4-048
NGUT-chr4-083	Ca21chr4	978050	978625	NOVEL-Ca21chr4-049
NGUT-chr4-084	Ca21chr4	997975	998500	NOVEL-Ca21chr4-050
NGUT-chr4-085	Ca21chr4	1031075	1031525	NOVEL-Ca21chr4-051
NGUT-chr4-086	Ca21chr4	1047125	1047350	
NGUT-chr4-087	Ca21chr4	1054850	1055075	TF4C225
NGUT-chr4-088	Ca21chr4	1101100	1101525	NOVEL-Ca21chr4-053
NGUT-chr4-089	Ca21chr4	1101700	1102050	ntar806
NGUT-chr4-090	Ca21chr4	1103150	1103350	ntar807
NGUT-chr4-091	Ca21chr4	1103450	1103850	NOVEL-Ca21chr4-054
NGUT-chr4-092	Ca21chr4	1107100	1107750	NOVEL-Ca21chr4-055
NGUT-chr4-093	Ca21chr4	1108050	1108450	TF4W6
NGUT-chr4-094	Ca21chr4	1114350	1114700	NOVEL-Ca21chr4-056
NGUT-chr4-095	Ca21chr4	1140100	1140350	
NGUT-chr4-096	Ca21chr4	1166225	1166675	NOVEL-Ca21chr4-058
NGUT-chr4-097	Ca21chr4	1177900	1178650	NOVEL-Ca21chr4-059
NGUT-chr4-098	Ca21chr4	1192625	1193350	NOVEL-Ca21chr4-060
NGUT-chr4-099	Ca21chr4	1194100	1195025	NOVEL-Ca21chr4-061
NGUT-chr4-100	Ca21chr4	1220000	1221000	NOVEL-Ca21chr4-062
NGUT-chr4-101	Ca21chr4	1230050	1230375	TF4W196
NGUT-chr4-102	Ca21chr4	1244475	1245275	NOVEL-Ca21chr4-063

NGUT-chr4-103	Ca21chr4	1260150	1261525	NOVEL-Ca21chr4-064/65
NGUT-chr4-104	Ca21chr4	1261700	1262025	
NGUT-chr4-105	Ca21chr4	1275050	1275725	ntar820
NGUT-chr4-106	Ca21chr4	1275950	1276250	
NGUT-chr4-107	Ca21chr4	1286750	1287400	NOVEL-Ca21chr4-066
NGUT-chr4-108	Ca21chr4	1295425	1295775	
NGUT-chr4-109	Ca21chr4	1381725	1381950	TF4C227
NGUT-chr4-110	Ca21chr4	1405050	1405200	
NGUT-chr4-111	Ca21chr4	1405500	1405600	
NGUT-chr4-112	Ca21chr4	1406550	1406800	NOVEL-Ca21chr4-069
NGUT-chr4-113	Ca21chr4	1440350	1440650	TF4C153
NGUT-chr4-114	Ca21chr4	1449475	1449900	NOVEL-Ca21chr4-070
NGUT-chr4-115	Ca21chr4	1452950	1453250	
NGUT-chr4-116	Ca21chr4	1453650	1454250	NOVEL-Ca21chr4-071
NGUT-chr4-117	Ca21chr4	1454375	1454525	TF4C40
NGUT-chr4-118	Ca21chr4	1456775	1457200	NOVEL-Ca21chr4-072
NGUT-chr4-119	Ca21chr4	1467275	1467775	TF4C7
NGUT-chr4-120	Ca21chr4	1470050	1470450	TF4W195
NGUT-chr4-121	Ca21chr4	1470575	1470900	TF4W188
NGUT-chr4-122	Ca21chr4	1488750	1489475	ntar836
NGUT-chr4-123	Ca21chr4	1520525	1522375	NOVEL-Ca21chr4-073
NGUT-chr4-124	Ca21chr4	1524900	1525075	
NGUT-chr4-125	Ca21chr4	1598425	1598850	NOVEL-Ca21chr4-074
NGUT-chr4-126	Ca21chr4	1602075	1602400	NOVEL-Ca21chr4-075
NGUT-chr4-127	Ca21chr4	1602400	1602850	ntar843
NGUT-chr5-001	Ca21chr5	3325	3525	TF5C21
NGUT-chr5-002	Ca21chr5	3600	3900	TF5C56
NGUT-chr5-003	Ca21chr5	30000	30225	TF5C57
NGUT-chr5-004	Ca21chr5	36100	36525	
NGUT-chr5-005	Ca21chr5	72500	72800	
NGUT-chr5-006	Ca21chr5	89000	89400	
NGUT-chr5-007	Ca21chr5	94225	94600	NOVEL-Ca21chr5-002
NGUT-chr5-008	Ca21chr5	101900	102100	TF5W172
NGUT-chr5-009	Ca21chr5	102600	102750	TF5C67
NGUT-chr5-010	Ca21chr5	114500	114875	
NGUT-chr5-011	Ca21chr5	124150	124350	ntar855
NGUT-chr5-012	Ca21chr5	136200	136450	TF5W32
NGUT-chr5-013	Ca21chr5	136575	137050	NOVEL-Ca21chr5-003
NGUT-chr5-014	Ca21chr5	150425	151000	TF5W3
NGUT-chr5-015	Ca21chr5	154500	154675	TF5C73
NGUT-chr5-016	Ca21chr5	155000	157750	NOVEL-Ca21chr5-005
NGUT-chr5-017	Ca21chr5	179150	179700	ntar859
NGUT-chr5-018	Ca21chr5	192375	193750	NOVEL-Ca21chr5-006
NGUT-chr5-019	Ca21chr5	205150	205550	
NGUT-chr5-020	Ca21chr5	209550	210100	
NGUT-chr5-021	Ca21chr5	228125	228900	NOVEL-Ca21chr5-007
NGUT-chr5-022	Ca21chr5	231300	231525	
NGUT-chr5-023	Ca21chr5	231700	232450	NOVEL-Ca21chr5-008
NGUT-chr5-024	Ca21chr5	232750	233350	NOVEL-Ca21chr5-009
NGUT-chr5-025	Ca21chr5	248900	249450	

NGUT-chr5-026	Ca21chr5	250750	251925	NOVEL-Ca21chr5-010/11
NGUT-chr5-027	Ca21chr5	255250	255800	
NGUT-chr5-028	Ca21chr5	256300	257000	NOVEL-Ca21chr5-013
NGUT-chr5-029	Ca21chr5	265300	265675	NOVEL-Ca21chr5-015
NGUT-chr5-030	Ca21chr5	280025	280325	TF5C35
NGUT-chr5-031	Ca21chr5	336900	337250	NOVEL-Ca21chr5-016
NGUT-chr5-032	Ca21chr5	351350	351800	NOVEL-Ca21chr5-017
NGUT-chr5-033	Ca21chr5	351850	352925	NOVEL-Ca21chr5-018
NGUT-chr5-034	Ca21chr5	369200	369675	ntar879
NGUT-chr5-035	Ca21chr5	380550	380950	
NGUT-chr5-036	Ca21chr5	406850	407325	
NGUT-chr5-037	Ca21chr5	419700	420400	
NGUT-chr5-038	Ca21chr5	448950	450000	ntar885
NGUT-chr5-039	Ca21chr5	465550	465825	ntar886
NGUT-chr5-040	Ca21chr5	465875	466150	NOVEL-Ca21chr5-019
NGUT-chr5-041	Ca21chr5	500325	500725	TF5C90
NGUT-chr5-042	Ca21chr5	516650	517125	NOVEL-Ca21chr5-021
NGUT-chr5-043	Ca21chr5	523300	523550	ntar896
NGUT-chr5-044	Ca21chr5	556875	557400	NOVEL-Ca21chr5-022
NGUT-chr5-045	Ca21chr5	561050	561100	TF5W38
NGUT-chr5-046	Ca21chr5	561300	561750	NOVEL-Ca21chr5-023
NGUT-chr5-047	Ca21chr5	580250	580300	
NGUT-chr5-048	Ca21chr5	593050	593250	TF5C97
NGUT-chr5-049	Ca21chr5	598700	598900	TF5C18
NGUT-chr5-050	Ca21chr5	599500	600225	NOVEL-Ca21chr5-024
NGUT-chr5-051	Ca21chr5	610625	611000	NOVEL-Ca21chr5-025
NGUT-chr5-052	Ca21chr5	636350	636825	
NGUT-chr5-053	Ca21chr5	637375	637800	NOVEL-Ca21chr5-031
NGUT-chr5-054	Ca21chr5	637850	638725	ntar906
NGUT-chr5-055	Ca21chr5	648050	648300	ntar907
NGUT-chr5-056	Ca21chr5	655000	655275	ntar909
NGUT-chr5-057	Ca21chr5	655400	655550	TF5C173
NGUT-chr5-058	Ca21chr5	656375	656500	
NGUT-chr5-059	Ca21chr5	667100	667425	TF5W14
NGUT-chr5-060	Ca21chr5	677925	678450	ntar911
NGUT-chr5-061	Ca21chr5	682025	682400	
NGUT-chr5-062	Ca21chr5	694725	696075	ntar913
NGUT-chr5-063	Ca21chr5	696550	696800	
NGUT-chr5-064	Ca21chr5	698850	699100	TF5C108
NGUT-chr5-065	Ca21chr5	707800	708350	ntar914
NGUT-chr5-066	Ca21chr5	708600	709450	ntar915
NGUT-chr5-067	Ca21chr5	712525	712800	ntar916
NGUT-chr5-068	Ca21chr5	722700	723125	TF5C15
NGUT-chr5-069	Ca21chr5	723225	723550	
NGUT-chr5-070	Ca21chr5	723900	724100	
NGUT-chr5-071	Ca21chr5	724350	724600	
NGUT-chr5-072	Ca21chr5	724675	725850	NOVEL-Ca21chr5-032/33
NGUT-chr5-073	Ca21chr5	750925	751050	
NGUT-chr5-074	Ca21chr5	751300	752175	NOVEL-Ca21chr5-034
NGUT-chr5-075	Ca21chr5	760075	760275	TF5W116

NGUT-chr5-076	Ca21chr5	773075	773675	NOVEL-Ca21chr5-035
NGUT-chr5-077	Ca21chr5	774200	774500	TF5C117
NGUT-chr5-078	Ca21chr5	775400	775900	NOVEL-Ca21chr5-036
NGUT-chr5-079	Ca21chr5	776100	776425	ntar918
NGUT-chr5-080	Ca21chr5	789575	790100	NOVEL-Ca21chr5-037
NGUT-chr5-081	Ca21chr5	790225	790850	NOVEL-Ca21chr5-038
NGUT-chr5-082	Ca21chr5	815525	816150	NOVEL-Ca21chr5-039
NGUT-chr5-083	Ca21chr5	819200	819800	ntar926
NGUT-chr5-084	Ca21chr5	837200	837450	ntar929
NGUT-chr5-085	Ca21chr5	851400	851600	TF5W120
NGUT-chr5-086	Ca21chr5	851900	852500	
NGUT-chr5-087	Ca21chr5	853200	853400	
NGUT-chr5-088	Ca21chr5	854375	854700	
NGUT-chr5-089	Ca21chr5	854950	855200	TF5C156
NGUT-chr5-090	Ca21chr5	861800	861950	TF5W125
NGUT-chr5-091	Ca21chr5	862250	862650	
NGUT-chr5-092	Ca21chr5	867300	867900	
NGUT-chr5-093	Ca21chr5	871450	871600	ntar932
NGUT-chr5-094	Ca21chr5	872800	873525	NOVEL-Ca21chr5-040
NGUT-chr5-095	Ca21chr5	885950	886450	NOVEL-Ca21chr5-041
NGUT-chr5-096	Ca21chr5	886550	886850	NOVEL-Ca21chr5-042
NGUT-chr5-097	Ca21chr5	890400	890700	ntar939
NGUT-chr5-098	Ca21chr5	893675	893850	
NGUT-chr5-099	Ca21chr5	894600	894950	
NGUT-chr5-100	Ca21chr5	897725	898300	
NGUT-chr5-101	Ca21chr5	898575	899100	ntar940
NGUT-chr5-102	Ca21chr5	922950	923525	
NGUT-chr5-103	Ca21chr5	950400	951525	
NGUT-chr5-104	Ca21chr5	968900	969425	
NGUT-chr5-105	Ca21chr5	992950	993375	NOVEL-Ca21chr5-044
NGUT-chr5-106	Ca21chr5	1000900	1001625	
NGUT-chr5-107	Ca21chr5	1029750	1030125	ntar949
NGUT-chr5-108	Ca21chr5	1030350	1030575	ntar950
NGUT-chr5-109	Ca21chr5	1052200	1052600	NOVEL-Ca21chr5-045
NGUT-chr5-110	Ca21chr5	1052750	1053325	NOVEL-Ca21chr5-046
NGUT-chr5-111	Ca21chr5	1077325	1077825	NOVEL-Ca21chr5-047
NGUT-chr5-112	Ca21chr5	1083500	1083600	ntar954
NGUT-chr5-113	Ca21chr5	1084350	1085250	NOVEL-Ca21chr5-048
NGUT-chr5-114	Ca21chr5	1085800	1086700	NOVEL-Ca21chr5-049
NGUT-chr5-115	Ca21chr5	1087075	1087350	ntar959
NGUT-chr5-116	Ca21chr5	1116900	1117100	
NGUT-chr5-117	Ca21chr5	1118100	1118700	NOVEL-Ca21chr5-050
NGUT-chr5-118	Ca21chr5	1136225	1136500	
NGUT-chr5-119	Ca21chr5	1181050	1181650	NOVEL-Ca21chr5-051
NGUT-chr5-120	Ca21chr5	1181475	1181650	TF5W29
NGUT-chr5-121	Ca21chr5	1183225	1183500	
NGUT-chr5-122	Ca21chr5	1185200	1185425	TF5C53
NGUT-chr6-001	Ca21chr6	2875	3475	ntar965
NGUT-chr6-002	Ca21chr6	35400	36375	NOVEL-Ca21chr6-001
NGUT-chr6-003	Ca21chr6	38450	38750	TF6W92

NGUT-chr6-004	Ca21chr6	89775	90125	ntar970
NGUT-chr6-005	Ca21chr6	106550	106950	NOVEL-Ca21chr6-002
NGUT-chr6-006	Ca21chr6	129000	129550	NOVEL-Ca21chr6-003
NGUT-chr6-007	Ca21chr6	145600	146175	NOVEL-Ca21chr6-004
NGUT-chr6-008	Ca21chr6	152275	152750	
NGUT-chr6-009	Ca21chr6	153425	153600	
NGUT-chr6-010	Ca21chr6	155250	155500	NOVEL-Ca21chr6-005
NGUT-chr6-011	Ca21chr6	155700	156200	NOVEL-Ca21chr6-006
NGUT-chr6-012	Ca21chr6	157000	158025	NOVEL-Ca21chr6-007
NGUT-chr6-013	Ca21chr6	162650	162875	NOVEL-Ca21chr6-008
NGUT-chr6-014	Ca21chr6	163875	164100	
NGUT-chr6-015	Ca21chr6	167950	168900	ntar974
NGUT-chr6-016	Ca21chr6	181650	181800	ntar977
NGUT-chr6-017	Ca21chr6	216775	216950	
NGUT-chr6-018	Ca21chr6	217175	217775	NOVEL-Ca21chr6-009
NGUT-chr6-019	Ca21chr6	223250	223825	NOVEL-Ca21chr6-010
NGUT-chr6-020	Ca21chr6	272900	273850	NOVEL-Ca21chr6-012
NGUT-chr6-021	Ca21chr6	286300	287200	NOVEL-Ca21chr6-013
NGUT-chr6-022	Ca21chr6	290775	290825	
NGUT-chr6-023	Ca21chr6	308625	309300	NOVEL-Ca21chr6-014
NGUT-chr6-024	Ca21chr6	309425	310625	NOVEL-Ca21chr6-015
NGUT-chr6-025	Ca21chr6	311725	311925	TF6W113
NGUT-chr6-026	Ca21chr6	312325	313300	NOVEL-Ca21chr6-016
NGUT-chr6-027	Ca21chr6	318725	319325	NOVEL-Ca21chr6-017
NGUT-chr6-028	Ca21chr6	327150	327850	NOVEL-Ca21chr6-018
NGUT-chr6-029	Ca21chr6	330475	331100	NOVEL-Ca21chr6-019
NGUT-chr6-030	Ca21chr6	349400	349825	
NGUT-chr6-031	Ca21chr6	360900	361350	
NGUT-chr6-032	Ca21chr6	362000	362925	NOVEL-Ca21chr6-020
NGUT-chr6-033	Ca21chr6	365300	365575	
NGUT-chr6-034	Ca21chr6	392950	393225	
NGUT-chr6-035	Ca21chr6	413175	413400	TF6W132
NGUT-chr6-036	Ca21chr6	435550	435825	TF6C124
NGUT-chr6-037	Ca21chr6	435875	436200	NOVEL-Ca21chr6-022
NGUT-chr6-038	Ca21chr6	456450	456900	
NGUT-chr6-039	Ca21chr6	483200	483325	
NGUT-chr6-040	Ca21chr6	484450	485000	NOVEL-Ca21chr6-023
NGUT-chr6-041	Ca21chr6	485175	485575	ntar1004
NGUT-chr6-042	Ca21chr6	485900	485950	
NGUT-chr6-043	Ca21chr6	487350	487700	
NGUT-chr6-044	Ca21chr6	488525	488725	TF6W64
NGUT-chr6-045	Ca21chr6	489000	489475	TF6C116
NGUT-chr6-046	Ca21chr6	498350	498750	TF6W122
NGUT-chr6-047	Ca21chr6	544200	545000	
NGUT-chr6-048	Ca21chr6	549450	549725	TF6W94
NGUT-chr6-049	Ca21chr6	550125	550800	
NGUT-chr6-050	Ca21chr6	550950	551325	
NGUT-chr6-051	Ca21chr6	552150	552400	TF6W128
NGUT-chr6-052	Ca21chr6	557650	558150	ntar1009
NGUT-chr6-053	Ca21chr6	595500	596050	NOVEL-Ca21chr6-025

NGUT-chr6-054	Ca21chr6	616575	617100	
NGUT-chr6-055	Ca21chr6	636975	637075	TF6W69
NGUT-chr6-056	Ca21chr6	665475	665700	
NGUT-chr6-057	Ca21chr6	680825	681325	ntar1018
NGUT-chr6-058	Ca21chr6	702550	702925	TF6C12
NGUT-chr6-059	Ca21chr6	706100	706600	NOVEL-Ca21chr6-026
NGUT-chr6-060	Ca21chr6	707100	708150	NOVEL-Ca21chr6-027/ntar1024
NGUT-chr6-061	Ca21chr6	708400	709200	NOVEL-Ca21chr6-028/29
NGUT-chr6-062	Ca21chr6	713600	714425	ntar1025
NGUT-chr6-063	Ca21chr6	736150	737225	NOVEL-Ca21chr6-030
NGUT-chr6-064	Ca21chr6	744700	744950	
NGUT-chr6-065	Ca21chr6	747800	748125	
NGUT-chr6-066	Ca21chr6	756125	756550	
NGUT-chr6-067	Ca21chr6	787225	788050	NOVEL-Ca21chr6-032
NGUT-chr6-068	Ca21chr6	790825	791350	
NGUT-chr6-069	Ca21chr6	812675	812925	
NGUT-chr6-070	Ca21chr6	824925	825600	NOVEL-Ca21chr6-033
NGUT-chr6-071	Ca21chr6	837275	837700	NOVEL-Ca21chr6-034
NGUT-chr6-072	Ca21chr6	860000	860450	
NGUT-chr6-073	Ca21chr6	862425	862925	NOVEL-Ca21chr6-035
NGUT-chr6-074	Ca21chr6	868675	868900	HOK6
NGUT-chr6-075	Ca21chr6	869525	870150	HOK6
NGUT-chr6-076	Ca21chr6	870375	871625	NOVEL-Ca21chr6-036
NGUT-chr6-077	Ca21chr6	872050	872600	HOK6
NGUT-chr6-078	Ca21chr6	872800	874150	NOVEL-Ca21chr6-037/HOK6
NGUT-chr6-079	Ca21chr6	874250	874550	NOVEL-Ca21chr6-038
NGUT-chr6-080	Ca21chr6	874850	875600	NOVEL-Ca21chr6-039
NGUT-chr6-081	Ca21chr6	898325	898500	
NGUT-chr6-082	Ca21chr6	910625	911525	ntar1041
NGUT-chr6-083	Ca21chr6	912550	912900	ntar1042
NGUT-chr6-084	Ca21chr6	913375	913525	TF6W84
NGUT-chr6-085	Ca21chr6	926500	927550	NOVEL-Ca21chr6-040
NGUT-chr6-086	Ca21chr6	927600	927875	NOVEL-Ca21chr6-041
NGUT-chr6-087	Ca21chr6	932925	933425	
NGUT-chr6-088	Ca21chr6	962400	963050	NOVEL-Ca21chr6-043
NGUT-chr6-089	Ca21chr6	964050	964350	TF6C99
NGUT-chr6-090	Ca21chr6	969550	970025	
NGUT-chr6-091	Ca21chr6	979150	979275	
NGUT-chr6-092	Ca21chr6	979425	980000	
NGUT-chr6-093	Ca21chr6	987200	987525	TF6W133
NGUT-chr6-094	Ca21chr6	996450	996800	
NGUT-chr6-095	Ca21chr6	1027250	1027400	
NGUT-chr7-001	Ca21chr7	108600	108775	
NGUT-chr7-002	Ca21chr7	110700	111600	NOVEL-Ca21chr7-001
NGUT-chr7-003	Ca21chr7	118200	120100	NOVEL-Ca21chr7-002
NGUT-chr7-004	Ca21chr7	121275	121700	T7W7
NGUT-chr7-005	Ca21chr7	122250	122625	T7W12
NGUT-chr7-006	Ca21chr7	129800	130775	NOVEL-Ca21chr7-004
NGUT-chr7-007	Ca21chr7	131125	131400	ntar1061
NGUT-chr7-008	Ca21chr7	136825	136875	

NGUT-chr7-009	Ca21chr7	164450	164625	TF7C28
NGUT-chr7-010	Ca21chr7	169000	169425	TF7C8
NGUT-chr7-011	Ca21chr7	169500	169825	TF7W61
NGUT-chr7-012	Ca21chr7	170050	170650	NOVEL-Ca21chr7-006
NGUT-chr7-013	Ca21chr7	180150	180950	NOVEL-Ca21chr7-007/TF7C133
NGUT-chr7-014	Ca21chr7	182650	183025	
NGUT-chr7-015	Ca21chr7	183350	183775	NOVEL-Ca21chr7-009
NGUT-chr7-016	Ca21chr7	184950	185250	NOVEL-Ca21chr7-010
NGUT-chr7-017	Ca21chr7	185450	186250	NOVEL-Ca21chr7-011
NGUT-chr7-018	Ca21chr7	189050	189400	NOVEL-Ca21chr7-012
NGUT-chr7-019	Ca21chr7	193400	193800	NOVEL-Ca21chr7-013
NGUT-chr7-020	Ca21chr7	193825	194325	NOVEL-Ca21chr7-014
NGUT-chr7-021	Ca21chr7	206650	206850	TF7W130
NGUT-chr7-022	Ca21chr7	211325	211500	ntar1074
NGUT-chr7-023	Ca21chr7	227125	227550	NOVEL-Ca21chr7-015
NGUT-chr7-024	Ca21chr7	227625	227900	ntar1076
NGUT-chr7-025	Ca21chr7	230275	230500	RB2-7a
NGUT-chr7-026	Ca21chr7	233700	234500	NOVEL-Ca21chr7-016
NGUT-chr7-027	Ca21chr7	237050	237625	HOK7
NGUT-chr7-028	Ca21chr7	238150	238500	HOK7
NGUT-chr7-029	Ca21chr7	239050	239600	NOVEL-Ca21chr7-017
NGUT-chr7-030	Ca21chr7	239975	240900	NOVEL-Ca21chr7-018
NGUT-chr7-031	Ca21chr7	241000	241375	HOK7
NGUT-chr7-032	Ca21chr7	241725	242475	TF7W14
NGUT-chr7-033	Ca21chr7	243100	243625	
NGUT-chr7-034	Ca21chr7	243700	243950	
NGUT-chr7-035	Ca21chr7	265275	265625	NOVEL-Ca21chr7-019
NGUT-chr7-036	Ca21chr7	280225	280300	TF7C72
NGUT-chr7-037	Ca21chr7	280900	280975	
NGUT-chr7-038	Ca21chr7	283625	283800	
NGUT-chr7-039	Ca21chr7	284850	285375	
NGUT-chr7-040	Ca21chr7	294200	294350	TF7C78
NGUT-chr7-041	Ca21chr7	294500	294750	NOVEL-Ca21chr7-020
NGUT-chr7-042	Ca21chr7	294975	295525	ntar1085
NGUT-chr7-043	Ca21chr7	297175	297250	TF7C137
NGUT-chr7-044	Ca21chr7	297700	298300	NOVEL-Ca21chr7-021
NGUT-chr7-045	Ca21chr7	298625	298850	NOVEL-Ca21chr7-022
NGUT-chr7-046	Ca21chr7	299550	299675	
NGUT-chr7-047	Ca21chr7	301450	302500	NOVEL-Ca21chr7-023
NGUT-chr7-048	Ca21chr7	315450	315825	
NGUT-chr7-049	Ca21chr7	316150	316700	
NGUT-chr7-050	Ca21chr7	318975	320050	
NGUT-chr7-051	Ca21chr7	322575	322950	ntar1089
NGUT-chr7-052	Ca21chr7	328675	329325	ntar1090
NGUT-chr7-053	Ca21chr7	350125	350400	TF7C89
NGUT-chr7-054	Ca21chr7	361150	361850	NOVEL-Ca21chr7-024
NGUT-chr7-055	Ca21chr7	361925	362200	NOVEL-Ca21chr7-025
NGUT-chr7-056	Ca21chr7	362250	362825	NOVEL-Ca21chr7-026
NGUT-chr7-057	Ca21chr7	367025	367700	
NGUT-chr7-058	Ca21chr7	379600	380000	ntar1096

NGUT-chr7-059	Ca21chr7	389025	390150	NOVEL-Ca21chr7-027
NGUT-chr7-060	Ca21chr7	409500	410400	NOVEL-Ca21chr7-028
NGUT-chr7-061	Ca21chr7	423725	424975	NOVEL-Ca21chr7-029
NGUT-chr7-062	Ca21chr7	443400	444975	NOVEL-Ca21chr7-030
NGUT-chr7-063	Ca21chr7	445150	445950	NOVEL-Ca21chr7-031
NGUT-chr7-064	Ca21chr7	447925	448075	
NGUT-chr7-065	Ca21chr7	448800	449525	
NGUT-chr7-066	Ca21chr7	451875	452025	
NGUT-chr7-067	Ca21chr7	452150	452400	TF7C38
NGUT-chr7-068	Ca21chr7	452500	452925	
NGUT-chr7-069	Ca21chr7	453100	453300	
NGUT-chr7-070	Ca21chr7	453450	453900	
NGUT-chr7-071	Ca21chr7	454225	454500	
NGUT-chr7-072	Ca21chr7	454600	454900	TF7C143
NGUT-chr7-073	Ca21chr7	478175	478675	NOVEL-Ca21chr7-032
NGUT-chr7-074	Ca21chr7	478725	479300	NOVEL-Ca21chr7-033
NGUT-chr7-075	Ca21chr7	479400	479725	TF7C23
NGUT-chr7-076	Ca21chr7	485400	485575	
NGUT-chr7-077	Ca21chr7	485825	485900	
NGUT-chr7-078	Ca21chr7	486000	486500	
NGUT-chr7-079	Ca21chr7	516375	517000	
NGUT-chr7-080	Ca21chr7	539725	540200	NOVEL-Ca21chr7-034
NGUT-chr7-081	Ca21chr7	544500	544900	NOVEL-Ca21chr7-035
NGUT-chr7-082	Ca21chr7	545300	545475	TF7W142
NGUT-chr7-083	Ca21chr7	554200	555950	NOVEL-Ca21chr7-036
NGUT-chr7-084	Ca21chr7	556550	560075	NOVEL-Ca21chr7-037
NGUT-chr7-085	Ca21chr7	567700	567850	TF7C100
NGUT-chr7-086	Ca21chr7	573375	573725	
NGUT-chr7-087	Ca21chr7	574100	574300	ntar1113
NGUT-chr7-088	Ca21chr7	574925	575200	NOVEL-Ca21chr7-038
NGUT-chr7-089	Ca21chr7	575325	575675	HOK7-b
NGUT-chr7-090	Ca21chr7	576300	576725	NOVEL-Ca21chr7-039
NGUT-chr7-091	Ca21chr7	576775	577275	HOK7-b
NGUT-chr7-092	Ca21chr7	577575	578200	HOK7-b
NGUT-chr7-093	Ca21chr7	578775	579850	NOVEL-Ca21chr7-040
NGUT-chr7-094	Ca21chr7	579975	580825	HOK7-b
NGUT-chr7-095	Ca21chr7	581225	581550	HOK7-b
NGUT-chr7-096	Ca21chr7	582900	583975	RB2-7b
NGUT-chr7-097	Ca21chr7	587225	587500	RB2-7b
NGUT-chr7-098	Ca21chr7	592250	592775	NOVEL-Ca21chr7-041
NGUT-chr7-099	Ca21chr7	606325	607150	NOVEL-Ca21chr7-042
NGUT-chr7-100	Ca21chr7	607350	607850	NOVEL-Ca21chr7-043
NGUT-chr7-101	Ca21chr7	608875	609125	
NGUT-chr7-102	Ca21chr7	611500	611825	
NGUT-chr7-103	Ca21chr7	613900	614750	NOVEL-Ca21chr7-044
NGUT-chr7-104	Ca21chr7	668100	668850	NOVEL-Ca21chr7-045
NGUT-chr7-105	Ca21chr7	673075	673725	
NGUT-chr7-106	Ca21chr7	692675	693300	ntar1129
NGUT-chr7-107	Ca21chr7	701500	702550	NOVEL-Ca21chr7-046
NGUT-chr7-108	Ca21chr7	722275	723175	NOVEL-Ca21chr7-047

NGUT-chr7-109	Ca21chr7	729450	730575	NOVEL-Ca21chr7-048
NGUT-chr7-110	Ca21chr7	731200	731600	TF7W25
NGUT-chr7-111	Ca21chr7	751800	752100	
NGUT-chr7-112	Ca21chr7	752950	753500	
NGUT-chr7-113	Ca21chr7	760800	761675	NOVEL-Ca21chr7-053
NGUT-chr7-114	Ca21chr7	761700	762150	
NGUT-chr7-115	Ca21chr7	819850	820025	
NGUT-chr7-116	Ca21chr7	821525	821950	
NGUT-chr7-117	Ca21chr7	839975	840525	NOVEL-Ca21chr7-055
NGUT-chr7-118	Ca21chr7	852525	852875	
NGUT-chr7-119	Ca21chr7	909625	909850	
NGUT-chr7-120	Ca21chr7	910000	912025	
NGUT-chr7-121	Ca21chr7	912100	913025	NOVEL-Ca21chr7-056
NGUT-chr7-122	Ca21chr7	914975	914525	
NGUT-chr7-123	Ca21chr7	916450	917350	
NGUT-chr7-124	Ca21chr7	917525	917900	
NGUT-chr7-125	Ca21chr7	922800	923700	
NGUT-chr7-126	Ca21chr7	943600	944000	
NGUT-chr7-127	Ca21chr7	944350	944625	TF7C10
NGUT-chr7-128	Ca21chr7	948800	948925	TF7C126
NGUT-chrR-001	Ca21chrR	950	1000	TFRW66
NGUT-chrR-002	Ca21chrR	3800	3950	
NGUT-chrR-003	Ca21chrR	35975	36325	
NGUT-chrR-004	Ca21chrR	113050	113800	NOVEL-Ca21chrR-003
NGUT-chrR-005	Ca21chrR	163375	162675	NOVEL-Ca21chrR-005
NGUT-chrR-006	Ca21chrR	204100	204400	ntar1170
NGUT-chrR-007	Ca21chrR	209300	209725	TFRW7
NGUT-chrR-008	Ca21chrR	211000	215300	NOVEL-Ca21chrR-006
NGUT-chrR-009	Ca21chrR	219650	220400	NOVEL-Ca21chrR-007
NGUT-chrR-010	Ca21chrR	226350	227000	NOVEL-Ca21chrR-008
NGUT-chrR-011	Ca21chrR	230900	231000	
NGUT-chrR-012	Ca21chrR	268300	269275	ntar1181/1182
NGUT-chrR-013	Ca21chrR	296775	297100	
NGUT-chrR-014	Ca21chrR	298550	299425	NOVEL-Ca21chrR-010
NGUT-chrR-015	Ca21chrR	300425	300625	TFRW89
NGUT-chrR-016	Ca21chrR	317325	317900	
NGUT-chrR-017	Ca21chrR	338525	339100	NOVEL-Ca21chrR-011
NGUT-chrR-018	Ca21chrR	345325	345475	
NGUT-chrR-019	Ca21chrR	351500	352050	NOVEL-Ca21chrR-012
NGUT-chrR-020	Ca21chrR	371500	371900	ntar1194
NGUT-chrR-021	Ca21chrR	403800	404325	NOVEL-Ca21chrR-013
NGUT-chrR-022	Ca21chrR	419550	420175	NOVEL-Ca21chrR-014
NGUT-chrR-023	Ca21chrR	426625	427250	NOVEL-Ca21chrR-015
NGUT-chrR-024	Ca21chrR	444425	444600	
NGUT-chrR-025	Ca21chrR	444650	444825	
NGUT-chrR-026	Ca21chrR	455600	455950	
NGUT-chrR-027	Ca21chrR	469500	469850	ntar1200
NGUT-chrR-028	Ca21chrR	515825	516300	NOVEL-Ca21chrR-017
NGUT-chrR-029	Ca21chrR	543450	543850	ntar1207
NGUT-chrR-030	Ca21chrR	544100	544400	NOVEL-Ca21chrR-019

NGUT-chrR-031	Ca21chrR	554725	555000	ntar1212
NGUT-chrR-032	Ca21chrR	555725	556450	ntar1215/1216
NGUT-chrR-033	Ca21chrR	573350	573750	NOVEL-Ca21chrR-022
NGUT-chrR-034	Ca21chrR	573925	574225	
NGUT-chrR-035	Ca21chrR	588000	588300	
NGUT-chrR-036	Ca21chrR	596625	596775	TFRC109
NGUT-chrR-037	Ca21chrR	599200	599700	
NGUT-chrR-038	Ca21chrR	603350	603700	ntar1219
NGUT-chrR-039	Ca21chrR	611075	612800	NOVEL-Ca21chrR-024
NGUT-chrR-040	Ca21chrR	623825	624650	ntar1226
NGUT-chrR-041	Ca21chrR	626375	628850	NOVEL-Ca21chrR-026
NGUT-chrR-042	Ca21chrR	650300	650800	NOVEL-Ca21chrR-027
NGUT-chrR-043	Ca21chrR	670700	671000	
NGUT-chrR-044	Ca21chrR	730025	730650	NOVEL-Ca21chrR-031
NGUT-chrR-045	Ca21chrR	736300	736900	NOVEL-Ca21chrR-032
NGUT-chrR-046	Ca21chrR	772800	773000	TFRC341
NGUT-chrR-047	Ca21chrR	776600	777000	NOVEL-Ca21chrR-033
NGUT-chrR-048	Ca21chrR	791525	791625	
NGUT-chrR-049	Ca21chrR	881875	882875	ntar1253/54
NGUT-chrR-050	Ca21chrR	883050	883625	ntar1255/56
NGUT-chrR-051	Ca21chrR	955250	955525	TFRW334
NGUT-chrR-052	Ca21chrR	993825	994800	
NGUT-chrR-053	Ca21chrR	1003600	1003900	
NGUT-chrR-054	Ca21chrR	1026200	1026750	NOVEL-Ca21chrR-037
NGUT-chrR-055	Ca21chrR	1042075	1042500	NOVEL-Ca21chrR-038
NGUT-chrR-056	Ca21chrR	1047200	1047450	TFRC137
NGUT-chrR-057	Ca21chrR	1047650	1047975	TFRW46
NGUT-chrR-058	Ca21chrR	1053900	1054100	NOVEL-Ca21chrR-039
NGUT-chrR-059	Ca21chrR	1062175	1062400	
NGUT-chrR-060	Ca21chrR	1062600	1062675	
NGUT-chrR-061	Ca21chrR	1068000	1068225	TFRW24
NGUT-chrR-062	Ca21chrR	1070300	1070500	NOVEL-Ca21chrR-040
NGUT-chrR-063	Ca21chrR	1090400	1091350	ntar1277
NGUT-chrR-064	Ca21chrR	1092950	1094075	NOVEL-Ca21chrR-042
NGUT-chrR-065	Ca21chrR	1094250	1094700	NOVEL-Ca21chrR-043
NGUT-chrR-066	Ca21chrR	1120650	1121100	NOVEL-Ca21chrR-044
NGUT-chrR-067	Ca21chrR	1137150	1137300	
NGUT-chrR-068	Ca21chrR	1141875	1142125	
NGUT-chrR-069	Ca21chrR	1144900	1145500	
NGUT-chrR-070	Ca21chrR	1150450	1151475	NOVEL-Ca21chrR-047
NGUT-chrR-071	Ca21chrR	1153875	1155400	TER1
NGUT-chrR-072	Ca21chrR	1156925	1157225	
NGUT-chrR-073	Ca21chrR	1191500	1192275	
NGUT-chrR-074	Ca21chrR	1192550	1193550	NOVEL-Ca21chrR-049
NGUT-chrR-075	Ca21chrR	1243200	1243625	NOVEL-Ca21chrR-050
NGUT-chrR-076	Ca21chrR	1259150	1260400	NOVEL-Ca21chrR-051
NGUT-chrR-077	Ca21chrR	1260975	1261525	
NGUT-chrR-078	Ca21chrR	1272875	1273950	NOVEL-Ca21chrR-053
NGUT-chrR-079	Ca21chrR	1277500	1277825	NOVEL-Ca21chrR-054
NGUT-chrR-080	Ca21chrR	1286300	1287200	NOVEL-Ca21chrR-055

NGUT-chrR-081	Ca21chrR	1288800	1289800	
NGUT-chrR-082	Ca21chrR	1305500	1306300	NOVEL-Ca21chrR-057
NGUT-chrR-083	Ca21chrR	1310550	1310950	ntar1301
NGUT-chrR-084	Ca21chrR	1311000	1312800	NOVEL-Ca21chrR-058
NGUT-chrR-085	Ca21chrR	1313125	1315075	
NGUT-chrR-086	Ca21chrR	1324300	1324950	NOVEL-Ca21chrR-059
NGUT-chrR-087	Ca21chrR	1345100	1345350	TFRW349
NGUT-chrR-088	Ca21chrR	1351850	1352625	NOVEL-Ca21chrR-060
NGUT-chrR-089	Ca21chrR	1352975	1353175	RPS-R
NGUT-chrR-090	Ca21chrR	1353950	1354325	ntar1305
NGUT-chrR-091	Ca21chrR	1355075	1357000	HOK-R
NGUT-chrR-092	Ca21chrR	1357700	1358750	CA3
NGUT-chrR-093	Ca21chrR	1358900	1359200	NOVEL-Ca21chrR-061
NGUT-chrR-094	Ca21chrR	1359275	1360575	NOVEL-Ca21chrR-062
NGUT-chrR-095	Ca21chrR	1362200	1362700	NOVEL-Ca21chrR-063
NGUT-chrR-096	Ca21chrR	1370800	1371650	NOVEL-Ca21chrR-065
NGUT-chrR-097	Ca21chrR	1372300	1372675	
NGUT-chrR-098	Ca21chrR	1374575	1375225	
NGUT-chrR-099	Ca21chrR	1396400	1397350	NOVEL-Ca21chrR-066
NGUT-chrR-100	Ca21chrR	1403850	1404450	NOVEL-Ca21chrR-067
NGUT-chrR-101	Ca21chrR	1404525	1404675	TFRW51
NGUT-chrR-102	Ca21chrR	1404825	1405850	NOVEL-Ca21chrR-068
NGUT-chrR-103	Ca21chrR	1407600	1408500	
NGUT-chrR-104	Ca21chrR	1411450	1411675	NOVEL-Ca21chrR-069
NGUT-chrR-105	Ca21chrR	1412675	1413850	NOVEL-Ca21chrR-070/71
NGUT-chrR-106	Ca21chrR	1414175	1414750	NOVEL-Ca21chrR-072
NGUT-chrR-107	Ca21chrR	1415075	1416775	NOVEL-Ca21chrR-073
NGUT-chrR-108	Ca21chrR	1417800	1417900	
NGUT-chrR-109	Ca21chrR	1422025	1424575	NOVEL-Ca21chrR-074
NGUT-chrR-110	Ca21chrR	1427325	1427975	
NGUT-chrR-111	Ca21chrR	1428275	1429275	
NGUT-chrR-112	Ca21chrR	1431850	1432200	ntar1321
NGUT-chrR-113	Ca21chrR	1470300	1470550	
NGUT-chrR-114	Ca21chrR	1521625	1522000	NOVEL-Ca21chrR-076
NGUT-chrR-115	Ca21chrR	1535900	1536800	NOVEL-Ca21chrR-077
NGUT-chrR-116	Ca21chrR	1578775	1579025	
NGUT-chrR-117	Ca21chrR	1584950	1585350	NOVEL-Ca21chrR-080
NGUT-chrR-118	Ca21chrR	1632600	1633100	
NGUT-chrR-119	Ca21chrR	1659850	1660500	NOVEL-Ca21chrR-082
NGUT-chrR-120	Ca21chrR	1688400	1688775	NOVEL-Ca21chrR-083
NGUT-chrR-121	Ca21chrR	1690075	1690400	
NGUT-chrR-122	Ca21chrR	1694175	1694450	ntar1350
NGUT-chrR-123	Ca21chrR	1704975	1706050	
NGUT-chrR-124	Ca21chrR	1716275	1716750	
NGUT-chrR-125	Ca21chrR	1717725	1718300	NOVEL-Ca21chrR-084
NGUT-chrR-126	Ca21chrR	1718800	1719675	NOVEL-Ca21chrR-085
NGUT-chrR-127	Ca21chrR	1720000	1720900	
NGUT-chrR-128	Ca21chrR	1729625	1729900	ntar1354
NGUT-chrR-129	Ca21chrR	1741350	1741550	TFRC187
NGUT-chrR-130	Ca21chrR	1741725	1742750	NOVEL-Ca21chrR-086

NGUT-chrR-131	Ca21chrR	1771550	1772050	NOVEL-Ca21chrR-087
NGUT-chrR-132	Ca21chrR	1809725	1810000	ntar1362
NGUT-chrR-133	Ca21chrR	1826000	1826350	
NGUT-chrR-134	Ca21chrR	1830425	1830800	NOVEL-Ca21chrR-088
NGUT-chrR-135	Ca21chrR	1831425	1832025	NOVEL-Ca21chrR-089
NGUT-chrR-136	Ca21chrR	1831900	1832025	TFRW282
NGUT-chrR-137	Ca21chrR	1855525	1855900	ntar1364
NGUT-chrR-138	Ca21chrR	1881350	1881575	ntar1365
NGUT-chrR-139	Ca21chrR	1883625	1884150	NOVEL-Ca21chrR-090
NGUT-chrR-140	Ca21chrR	1884875	1885250	ntar1366
NGUT-chrR-141	Ca21chrR	1885550	1886500	TFRC2
NGUT-chrR-142	Ca21chrR	1888500	1889550	NOVEL-Ca21chrR-092
NGUT-chrR-143	Ca21chrR	1889800	1890100	NOVEL-Ca21chrR-093
NGUT-chrR-144	Ca21chrR	1890775	1897400	NOVEL-Ca21chrR-094/95
NGUT-chrR-145	Ca21chrR	1938675	1938975	ntar1372
NGUT-chrR-146	Ca21chrR	1939125	1939925	NOVEL-Ca21chrR-097
NGUT-chrR-147	Ca21chrR	1999300	2000025	NOVEL-Ca21chrR-098
NGUT-chrR-148	Ca21chrR	2009700	2010675	NOVEL-Ca21chrR-099
NGUT-chrR-149	Ca21chrR	2010975	2011625	
NGUT-chrR-150	Ca21chrR	2012275	2014100	NOVEL-Ca21chrR-100
NGUT-chrR-151	Ca21chrR	2041700	2042400	ntar1386
NGUT-chrR-152	Ca21chrR	2058875	2059150	TFRW221
NGUT-chrR-153	Ca21chrR	2061700	2061800	
NGUT-chrR-154	Ca21chrR	2062825	2063300	ntar1391
NGUT-chrR-155	Ca21chrR	2076375	2077300	ntar1394.1
NGUT-chrR-156	Ca21chrR	2077375	2078025	ntar1394.2
NGUT-chrR-157	Ca21chrR	2083450	2083950	NOVEL-Ca21chrR-103
NGUT-chrR-158	Ca21chrR	2084175	2084525	NOVEL-Ca21chrR-104
NGUT-chrR-159	Ca21chrR	2085075	2085450	NOVEL-Ca21chrR-105
NGUT-chrR-160	Ca21chrR	2085800	2086400	TFRW269
NGUT-chrR-161	Ca21chrR	2087500	2087925	ntar1400
NGUT-chrR-162	Ca21chrR	2099825	2100350	TFRW324
NGUT-chrR-163	Ca21chrR	2103850	2104225	NOVEL-Ca21chrR-107
NGUT-chrR-164	Ca21chrR	2121775	2122300	NOVEL-Ca21chrR-108
NGUT-chrR-165	Ca21chrR	2132675	2132950	
NGUT-chrR-166	Ca21chrR	2154975	2155575	TFRW255
NGUT-chrR-167	Ca21chrR	2169775	2170600	NOVEL-Ca21chrR-109
NGUT-chrR-168	Ca21chrR	2170900	2171250	NOVEL-Ca21chrR-110
NGUT-chrR-169	Ca21chrR	2203125	2203175	TFRW231
NGUT-chrR-170	Ca21chrR	2205275	2205550	
NGUT-chrR-171	Ca21chrR	2226925	2227300	
NGUT-chrR-172	Ca21chrR	2240500	2240775	TFRC27
NGUT-chrR-173	Ca21chrR	2243450	2243725	ntar1410

Supplementary table S3: CDR Induced genes. Genes that were found significantly overexpressed in Gu5 by Deseq and EdgeR. Log2(ratios) are indicated, together with CGD descriptions. The CDR isolates column indicate the number of different isolates in which the gene was previously found to be overexpressed, based on the studies of Liu et al., 2007 and Znaidi et al., 2006.

ID	Name	log2(Gu5/Gu4)	CDR isolates	ORF Status	Description
NGUT-chr5-037		23,1		NA	5' of TAC1
orf19.5958	CDR2	4,9	4	verified	Multidrug transporter, ATP-binding cassette (ABC) superfamily; transports phospholipids, in-to-out direction; overexpressed in azole-resistant isolates; repressed in young biofilms
orf19.5290		4,1		uncharacterised	Hap43p-induced gene; transcription is negatively regulated by Sfu1p
orf19.1430		3,9		uncharacterised	none
orf19.6864		3,8		uncharacterised	Has domain(s) with predicted ubiquitin-protein ligase activity, role in protein ubiquitination and ubiquitin ligase complex localization
orf19.4459		3,0	4	verified	Protein similar to S. cerevisiae Ynl234wp, which is a putative heme-binding stress-related protein; transposon mutation affects filamentous growth
orf19.23	RTA3	2,8	4	uncharacterised	Similar to S. cerevisiae Rta1p (role in 7-aminocholesterol resistance) and Rsb1p (flippase); putative membrane protein; putative drug-responsive regulatory site; induced by fluphenazine, estradiol, ketoconazole, caspofungin
NOVEL-Ca21chr4-073		2,5		NA	5' of CZF1
NGUT-chr2-038		2,4		NA	5' of ADAEC

orf19.6000	CDR1	2,3	4	verified	Multidrug transporter of ATP-binding cassette (ABC) superfamily; transports phospholipids in an in-to-out direction; transcription induced by beta-estradiol, progesterone, corticosteroid, or cholesterol; repressed in young biofilms
orf19.1438		2,3		uncharacterised	Protein with homology to NADH dehydrogenase; regulated by Sef1p-, Sfu1p-, and Hap43p
orf19.344		2,3	3	uncharacterised	Predicted ORF in Assemblies 19, 20 and 21; increased transcription is observed upon fluphenazine treatment or in an azole-resistant strain that overexpresses CDR1 and CDR2; possibly transcriptionally regulated by Tac1p
orf19.5713	YMX6	2,2	4	uncharacterised	Putative NADH dehydrogenase; macrophage-downregulated gene; induced by nitric oxide
orf19.7585	INO1	2,1	3	verified	Inositol-1-phosphate synthase; inositol biosynthesis; antigenic in human; repressed by farnesol in biofilm or by caspofungin; regulated during biofilm, planktonic growth; upstream inositol/choline regulatory element; glycosylation predicted
orf19.6501		2,1	3	uncharacterised	none
orf19.3374	ECE1	2,0		verified	Hyphal-specific protein; expression increases with extent of cell elongation; regulated by Rfg1p, Nrg1p, Tup1p, Cph1p, Efg1p, Hog1p, farnesol, phagocytosis; fluconazole-induced; may contribute to biofilm formation

orf19.3337		2,0		uncharacterised	Late-stage biofilm-induced gene
orf19.3338		1,9	2	uncharacterised	none
orf19.3160	HSP12	1,9	4	uncharacterised	Heat-shock protein; induced upon osmotic/oxidative/cadmium stress, fluphenazine treatment, low iron, CDR1 and CDR2 overexpression, or ssn6 or ssk1 homozygous null mutation; repressed by Hog1p, flucytosine, elevated CO2
orf19.3127	CZF1	1,9		verified	Transcriptional regulator of white-opaque switching frequency; hyphal growth regulator; C-terminal zinc finger and central Glu-rich region; expression in S. cerevisiae causes dominant-negative inhibition of pheromone response
orf19.3548.1	WH11	1,8		verified	Cytoplasmic protein expressed specifically in white phase yeast cells; expression in opaque cells increases virulence and frequency of opaque-to-white switching; null mutant has wild-type switching; Hap43p, hypoxia and ketoconazol induced
orf19.86		1,8	2	uncharacterised	Putative glutathione peroxidase; peroxide-induced; induced in response to peroxide, exposure to neutrophils and macrophage blood fractions; downregulated during infection of macrophages
orf19.4216		1,8		uncharacterised	Putative Products heat shock protein; decreased expression in hyphae compared to yeast-form cells; transcription is increased in populations of cells exposed to fluconazole over multiple generations

orf19.6202	RBT4	1,8	3	verified	Protein similar to plant pathogenesis-related proteins; required for virulence in mouse systemic and rabbit corneal infections; not required for filamentation; mRNA binds to She3p and is localized to hyphal tips; Hap43p-induced gene
orf19.3188	TAC1	1,8	4	verified	Transcriptional activator of drug-responsive genes including CDR1 and CDR2; has Zn(2)-Cys(6) binuclear cluster; binds DRE element; gene in zinc cluster region near MTL locus; resequencing indicates that TAC1 spans orf19.3188 and orf19.3189
orf19.4056	BRG1	1,7	3	verified	Putative DNA-binding transcription factor; similar to S. cerevisiae Gat2p; transposon mutation affects filamentous growth; Hap43p-repressed gene; late-stage biofilm-induced
orf19.4531		1,7	4	uncharacterised	Putative PDR-subfamily ABC transporter
orf19.2568	IFU5	1,6	4	uncharacterised	Predicted membrane protein; estradiol-induced; increased transcription associated with CDR1 and CDR2 overexpression or fluphenazine treatment; putative drug-responsive regulatory site; similar to S. cerevisiae Wwm1p; Hap43p-repressed gene
orf19.2344	ASR1	1,6	1	uncharacterised	Putative heat shock protein; transcription regulated by cAMP, osmotic stress, ciclopirox olamine, ketoconazole; negatively regulated by Cyr1p, Ras1p; colony morphology-related regulation by Ssn6p; stationary phase enriched; Hap43p-induced
orf19.938		1,5		uncharacterised	none

orf19.7310		1,4	4	uncharacterised	Protein similar to S. cerevisiae Gin3p; transcription is upregulated in response to treatment with ciclopirox olamine; positively regulated by Sfu1p; Hog1p, fluconazole-downregulated; Hap43p-induced
orf19.5785		1,4		uncharacterised	Predicted ORF in Assemblies 19, 20 and 21; greater mRNA abundance observed in a cyr1 or ras1 homozygous null mutant than in wild type; induced by nitric oxide
orf19.4477	CSH1	1,4	4	verified	Aldo-keto reductase family member, similar to aryl alcohol dehydrogenases; role in fibronectin adhesion, cell surface hydrophobicity; regulated by temperature, growth phase, benomyl, macrophage interaction; azole resistance associated
orf19.2849	AQY1	1,3	4	verified	Aquaporin water channel; mutant has increased resistance to osmotic shock; required for wild-type tolerance of freezing; not required for virulence in a mouse model of systemic infection; flucytosine repressed; biofilm-induced gene
orf19.7284	ASR2	1,3	4	uncharacterised	Gene regulated by cAMP and by osmotic stress; greater mRNA abundance observed in a cyr1 or ras1 homozygous null mutant than in wild type; stationary phase enriched protein
orf19.2285		1,3	3	verified	Increased transcription is observed upon benomyl treatment

orf19.6026	ERG2	1,2	3	verified	C-8 sterol isomerase; enzyme of ergosterol biosynthesis pathway; converts fecosterol to episterol; mutant is hypersensitive to multiple drugs; ketoconazole-induced
orf19.6556		1,2		uncharacterised	Late-stage biofilm-induced gene
orf19.5879		1,2	2	uncharacterised	Has domain(s) with predicted nucleotide binding, oxidoreductase activity and role in oxidation-reduction process
orf19.896	CHK1	1,2	4	verified	Histidine kinase involved in two-component signaling pathway, regulates cell wall biosynthesis; disruption impairs hyphal growth, decreases virulence in mouse intravenous (but not rat vaginal) infection and increases rate of phagocytosis
orf19.2244		1,1	4	uncharacterised	Similar to oxidoreductases and to S. cerevisiae Yjr096wp; transcription is negatively regulated by Sfu1p; increased transcription upon benomyl treatment; decreased expression in an ssr1 null mutant; Hap43p-repressed gene
orf19.4255	ECM331	1,1	3	verified	GPI-anchored protein; mainly at plasma membrane, also at cell wall; caspofungin induced; Plc1p-regulated; repressed by Rim101p, Hog1p; colony morphology-related regulation by Ssn6p; induced by ketoconazole and by hypoxia; Hap43p-induced

orf19.1862		1,1	3	uncharacterised	Possible stress protein; increased transcription associated with CDR1 and CDR2 overexpression or fluphenazine treatment; transcription regulated by Sfu1p, Nrg1p, Tup1p; stationary phase enriched protein
orf19.1027	PDR16	1,1	3	verified	Phosphatidylinositol transfer protein; increased transcription correlates with CDR1 and CDR2 overexpression and azole resistance; induced by fluphenazine, 17-beta-estradiol, ethynyl estradiol, nitric oxide; farnesol-downregulated in biofilm
TF3W223		1,1		NA	5' of orf19.6713
orf19.1783	YOR1	1,1		verified	Protein similar to S. cerevisiae Yor1p, which is a plasma membrane transporter of the ATP-binding cassette (ABC) family involved in resistance to aureobasidin A; transcription is specific to white cell type
orf19.4082	DDR48	1,1	3	verified	Immunogenic stress-associated protein; regulated by filamentous growth pathways; induced by benomyl, caspofungin, ketoconazole or in azole-resistant strain; Hog1p, farnesol, alkaline downregulated; stationary phase enriched; biofilm-induced
orf19.4476		1,0	2	uncharacterised	Predicted ORF in Assemblies 19, 20 and 21; transcript induced by ketoconazole
orf19.6007		1,0		uncharacterised	Predicted ORF in Assemblies 19, 20 and 21; decreased expression in response to prostaglandins
orf19.7380		1,0		uncharacterised	Has domain(s) with predicted nucleic acid binding, nucleotide binding activity

orf19.2531	CSP37	0,9	4	verified	Plasma membrane, hyphal cell wall protein; role in progression of murine systemic infection; predicted P-loop, divalent cation binding, N-glycosylation sites; expressed in yeast and hyphae; hyphal downregulated; stationary-phase enriched
orf19.1150.1		0,9		uncharacterised	none
orf19.467		0,9		uncharacterised	none
orf19.6586		0,9	2	verified	Late-stage biofilm-induced gene; increased transcription observed upon benomyl treatment or in an azole-resistant strain that overexpresses MDR1; shows colony morphology-related regulation by Ssn6p; induced by nitric oxide; Hap43p-repressed
orf19.3434		0,9		uncharacterised	none
orf19.3969	SFL2	0,9		verified	Probable transcription factor required for filamentous growth; required for virulence in an RHE model but not in mouse systemic infection; upregulated upon RHE infection but C. dubliniensis ortholog is not upregulated
orf19.4246		0,9	2	verified	Protein with similarity to S. cerevisiae Ykr070wp; transposon mutation affects filamentous growth; Hog1p-downregulated; shows colony morphology-related gene regulation by Ssn6p; induced during cell wall regeneration; possibly essential gene

orf19.5636	RBT5	0,9	3	verified	GPI-anchored cell wall protein involved in hemoglobin utilization; transcript regulated by Rfg1p, Rim101p, Tbf1p, iron; repressed by Sfu1p, Hog1p, Tup1p; induced by serum, alkaline pH, ketoconazole, ciclopirox olamine, Hap43p, biofilm
NOVEL-Ca21chr3-056	TF3W225	0,9		NA	5' of AAF1
orf19.3190	HAL9	0,9	4	uncharacterised	Protein with Zn(2)-Cys(6) binuclear cluster; gene in zinc cluster region of Chr. 5; transcriptionally activated by Mnl1p in weak acid; similar to S. cerevisiae Hal9p, which is a putative transcription factor involved in salt tolerance
orf19.5242	CDC6	0,9		verified	Putative ATP-binding protein with a predicted role in DNA replication; member of conserved Mcm1p regulon; periodic mRNA expression, peak at cell-cycle M/G1 phase
orf19.6420	PGA13	0,9	1	uncharacterised	Putative adhesin-like GPI-anchored protein; similar to mucins; induced during cell wall regeneration and during core caspofungin response; regulated by Tsa1p, Tsa1Bp in minimal media at 37 deg and by Cyr1p, Nrg1p, Tup1p, Rlm1p, Hap43p
orf19.4273		0,9		uncharacterised	Hap43p-induced gene
orf19.5070		0,9	1	uncharacterised	Similar to cell-wall mannoproteins; transcriptionally regulated by iron; expression greater in low iron; greater mRNA abundance observed in cyr1 homozygous null mutant than in wild type; regulated by osmotic and oxidative stress via Hog1p

orf19.7472	IFF4	0,8	2	verified	Adhesin-like cell surface protein; putative GPI-anchor; null mutant germ tubes show decreased adhesion to plastic substrate; not essential for viability; Hap43p-repressed gene
orf19.5741	ALS1	0,8		verified	Adhesin; ALS family of cell-surface glycoproteins; adhesion, virulence roles; immunoprotective; band at hyphal base; amyloid domain; biofilm-induced; Rfg1p, Ssk1p; strain background affects expression; N-term binds fucose-containing glycans
orf19.7166		0,8	4	uncharacterised	Predicted ORF in Assemblies 19, 20 and 21; increased transcription is observed in an azole-resistant strain that overexpresses MDR1; transcriptionally activated by Mnl1p under weak acid stress
orf19.2613	ECM4	0,8	3	verified	Cytoplasmic glutathione S-transferase; transcription regulated by Nrg1p, Tup1p; induced in core stress response, in cyr1 or ras1 null mutant (yeast or hyphal cells); transposon mutation affects filamentous growth; stationary phase enriched
orf19.5257	LCB4	0,8	4	uncharacterised	Putative sphingosine kinase; expression is Tac1p-regulated

orf19.2896	SOU1	0,8	4	verified	Enzyme involved in utilization of L-sorbose; has sorbitol dehydrogenase, fructose reductase, and sorbose reductase activities; NAD-binding site motif; transcriptional regulation affected by chromosome 5 copy number; Hap43p-induced gene
orf19.2280	ZCF10	0,8	4	uncharacterised	Putative transcription factor with zinc cluster DNA-binding motif
orf19.2952	EXG2	0,8	3	verified	GPI-anchored cell wall protein, similar to S. cerevisiae exo-1,3-beta-glucosidase Exg2p; predicted Kex2p substrate; induced during cell wall regeneration; possibly an essential gene, disruptants not obtained by UAU1 method; Hap43p-repressed
orf19.35.1		0,8		uncharacterised	none
orf19.674		0,8		uncharacterised	Has domain(s) with predicted nucleotide binding, nucleic acid binding, zinc ion binding activity
orf19.2881	MNN4	0,8		verified	Protein required for normal mannosylphosphorylation of oligosaccharides linked to cell wall proteins, not required for virulence or kidney colonization in mouse systemic infection or for normal interaction with macrophages
orf19.3461		0,7	2	uncharacterised	Predicted ORF in Assemblies 19, 20 and 21; oxidative stress-induced via Cap1p; transcription is induced in response to alpha pheromone in SpiderM medium
orf19.744	GDB1	0,7	4	uncharacterised	Putative glycogen debranching enzyme; expression is regulated upon white-opaque switching; regulated by Nrg1p, Tup1p

orf19.93		0,7		uncharacterised	Putative mitochondrial intermembrane space protein; shows colony morphology-related gene regulation by Ssn6p; regulated by Sef1p-, Sfu1p-, and Hap43p
orf19.1562		0,7	1	uncharacterised	Late-stage biofilm-induced gene; transcription is repressed in response to alpha pheromone in SpiderM medium
orf19.6888		0,7		uncharacterised	Protein with Gal4p-like DNA-binding domain; gene transcription regulated by Mig1p and Tup1p
orf19.740	HAP41	0,7		verified	Hap43p-repressed gene; not required for response to low iron; transcriptionally activated by Mnl1p under weak acid stress
orf19.2724		0,7	1	uncharacterised	Hap43p-repressed gene; late-stage biofilm-induced
orf19.1267		0,7	4	uncharacterised	Has domain(s) with predicted unfolded protein binding, heat shock protein binding activity and role in protein folding
orf19.4716	GDH3	0,7		verified	NADP-glutamate dehydrogenase; hyphal downregulated; regulated by Nrg1p, Plc1p; Efg1p-downregulated; upregulated by Rim101p at pH 8; ciclopirox, ketoconazole induced; repressed by hypoxia; protein present in exponential and stationary phases
orf19.7214		0,7	3	uncharacterised	Putative glucan 1,3-beta-glucosidase; regulated by Nrg1p, Tup1p and possibly Tac1p; induced upon biofilm formation, nitric oxide and during cell wall regeneration; stationary phase enriched protein; possibly an essential gene

orf19.3932		0,7	1	uncharacterised	Stationary phase enriched protein; induced in core caspofungin response; induced by nitric oxide independent of Yhb1p; repressed in a ssr1 null mutant; induced by ketoconazole and by hypoxia
orf19.7218	RBE1	0,7	4	verified	Cell wall protein; transcript negatively regulated by Rim101p, Efg1p, Ssn6p, alkaline conditions; signal sequence, O-glycosylation; no GPI anchor predicted; ketoconazol upregulated; biofilm-induced, regulated by Sef1p, Sfu1p, Hap43p
orf19.4393	CIT1	0,7		verified	Citrate synthase; soluble in hyphae; expression greater in high iron; upregulated by phagocytosis, biofilm growth; Hog1p-downregulated; Efg1p-regulated under yeast, not hyphal growth conditions; present in exponential and stationary phase
orf19.3769		0,7		uncharacterised	Ortholog(s) have zinc ion transmembrane transporter activity, role in zinc ion transport and endoplasmic reticulum localization
orf19.868	ADAEC	0,6		uncharacterised	Protein of unknown function; transcription is specific to white cell type
orf19.6713		0,6		uncharacterised	Has domain(s) with predicted nucleic acid binding, zinc ion binding activity and intracellular localization
orf19.2846		0,6		uncharacterised	Hap43p-repressed gene; induced in core caspofungin response; transcription regulated upon yeast-hyphal switch

orf19.558	GUT1	0,6		uncharacterised	Putative glycerol kinase; downregulated upon adherence to polystyrene; greater mRNA abundance observed in a cyr1 homozygous null mutant than in wild type
orf19.6968		0,6		uncharacterised	Predicted ORF in Assemblies 19, 20 and 21; Hog1p-downregulated
orf19.4898		0,6	4	uncharacterised	Putative protein of unknown function; increased expression in response to prostaglandins
orf19.2296		0,6	4	uncharacterised	Protein with similarity to mucins; ketoconazole-induced; fluconazole-downregulated; mRNA abundance increased in a cyr1 mutant than in wild type; colony morphology-related gene regulation by Ssn6p; late-stage biofilm-induced
orf19.5805	DLD1	0,6		verified	Putative D-lactate dehydrogenase; transcription is specific to white cell type; shows colony morphology-related gene regulation by Ssn6p; transcription is upregulated in both intermediate and mature biofilms; Hap43p-repressed gene
orf19.1887	YEH1	0,6	4	uncharacterised	Ortholog(s) have sterol esterase activity, role in sterol metabolic process and lipid particle, integral to membrane localization
orf19.6527		0,6		uncharacterised	Predicted ORF in Assemblies 19, 20 and 21; shows colony morphology-related gene regulation by Ssn6p; transcriptionally activated by Mnl1p under weak acid stress; possibly an essential gene, disruptants not obtained by UAU1 method

orf19.3644		0,6	4	uncharacterised	Predicted ORF in Assemblies 19, 20 and 21; greater mRNA abundance observed in a cyr1 homozygous null mutant than in wild type
orf19.3893	SCW11	0,6	1	verified	Cell wall protein; transcription decreased in mutant lacking ACE2; downregulated in core caspofungin response; expression greater in high iron; possibly an essential gene, disruptants not obtained by UAU1 method; planktonic growth-induced
orf19.829	SCH9	0,6		verified	Protein kinase involved in growth control, normal cell size, resistance to rapamycin and cations, chlamydospore formation, filamentous growth under some conditions, and virulence; prevents hypha formation under hypoxia at high CO2 levels
orf19.2726		0,6	4	uncharacterised	Putative plasma membrane protein; Plc1p-regulated
orf19.4688	DAG7	0,6	2	verified	Secretory protein; alpha-factor induced gene; mutation confers hypersensitivity to toxic ergosterol analog; a-specific transcription; planktonic growth-induced; fluconazole-induced
orf19.6724	FUM12	0,6		uncharacterised	Putative fumarate hydratase, enzyme of citric acid cycle; fluconazole-downregulated; downregulated by Efg1p; transcriptionally regulated by iron; expression greater in high iron; protein present in exponential and stationary growth phase
orf19.2959.1		0,6		uncharacterised	Gene induced by hypoxia and ketoconazole
orf19.7341.1		0,6		uncharacterised	none

orf19.3007.2		0,6	4	uncharacterised	none
orf19.5843	SRR1	0,6		verified	Response regulator of a two-component system involved in stress, morphogenesis, virulence; late-stage biofilm-induced ; Plc1p-regulated; greater mRNA abundance observed in a cyr1 homozygous null mutant than in wild type
orf19.1439	IPK1	0,6		uncharacterised	Ortholog(s) have inositol pentakisphosphate 2-kinase activity, role in nuclear-transcribed mRNA catabolic process, non-stop decay, inositol phosphate biosynthetic process and nucleus localization
orf19.251	HSP31	0,6	2	verified	ThiJ/PfpI protein; binds human immunoglobulin E; 2 N-glycosylation motifs; alkaline, fluconazole, Hog1p-downregulated; induced in core stress response or by oxidative stress (via Cap1p); induced by hypoxia, Hap43p; stationary-phase enriched
orf19.1290	XKS1	0,6	4	uncharacterised	Putative xylulokinase; Hap43p-repressed gene; increased expression in response to prostaglandins
orf19.5753	HGT10	0,6		verified	Glycerol permease involved in glycerol uptake; member of the major facilitator superfamily; induced by osmotic stress, at low glucose in rich media, during cell wall regeneration; 12 membrane spans; Hap43p-induced gene
orf19.2790	SWD2	0,5	4	uncharacterised	Ortholog(s) have histone methyltransferase activity (H3-K4 specific) activity
orf19.4347		0,5	2	uncharacterised	Putative serine/threonine protein kinase; Hog1p-induced

orf19.4532		0,5		uncharacterised	Protein of unknown function; present in exponential and stationary growth phase yeast cultures
orf19.3104	YDC1	0,5	4	uncharacterised	Protein with Mob2p-dependent hyphal regulation; transcription is regulated by Nrg1p and Mig1p; Hap43p-repressed gene
orf19.2175		0,5	3	uncharacterised	Putative mitochondrial cell death effector; induced by nitric oxide
orf19.3045		0,5	3	uncharacterised	Predicted ORF in Assemblies 19, 20 and 21; virulence-group-correlated expression
orf19.3675	GAL7	0,5		uncharacterised	Putative galactose-1-phoshphate uridyl transferase; downregulated by hypoxia, upregulated by ketoconazole; macrophage/pseudohyphal-repressed
orf19.4679	AGP2	0,5	1	uncharacterised	Amino acid permease; hyphal downregulated; regulated upon white-opaque switching; induced in core caspofungin response, during cell wall regeneration, or by flucytosine; fungal-specific; regulated by Sef1p-, Sfu1p-, and Hap43p
orf19.1944	GPR1	0,5		verified	Plasma membrane G-protein-coupled receptor of the cAMP-PKA pathway; required for wild-type hyphal growth; reports differ on role in cAMP-mediated glucose signaling; Gpr1p C terminus binds Gpa2p; regulates HWP1 and ECE1; biofilm-induced
orf19.3133	GUT2	0,5	2	verified	Glycerol-3-phosphate dehydrogenase; Plc1p-regulated; transcription is upregulated in both intermediate and mature biofilms

orf19.5102	PLB5	0,5	3	verified	Putative GPI-linked phospholipase B, fungal-specific (no mammalian homolog); null mutation eliminates cell-associated phospholipase A2 activity and attenuates virulence; fluconazole-repressed
orf19.6445	ECI1	0,5	3	verified	Protein similar to S. cerevisiae Eci1p, which is involved in fatty acid oxidation; transposon mutation affects filamentous growth; expression is regulated upon white-opaque switching
orf19.6659	GAP6	0,5	3	verified	Broad-specificity amino acid permease; Plc1p-regulated; Gcn4p-regulated; fungal-specific (no human or murine homolog)
orf19.1395		0,5	3	uncharacterised	Ortholog(s) have inorganic phosphate transmembrane transporter activity, role in transmembrane transport, phosphate ion transport and mitochondrion localization
orf19.2706	CRH11	0,5		verified	GPI-anchored cell wall transglycosylase, putative ortholog of S. cerevisiae Crh1p; predicted glycosyl hydrolase domain; similar to Csf4p and to antigenic A. fumigatus Aspf9; predicted Kex2p substrate; caspofungin-induced
orf19.6608		0,5		uncharacterised	Predicted ORF in Assemblies 19, 20 and 21; downregulation correlates with clinical development of fluconazole resistance
orf19.3869		0,5		uncharacterised	Predicted ORF in Assemblies 19, 20 and 21; regulated by Tsa1p, Tsa1Bp in minimal media at 37 deg; shows colony morphology-related gene regulation by Ssn6p

orf19.4416	VPS13	0,4		uncharacterised	Putative vacuolar protein sorting-associated protein; gene used for multilocus sequence typing
orf19.6229	CAT1	0,4	1	verified	Catalase; resistance to oxidative stress, neutrophils, peroxide; role in virulence; regulated by iron, ciclopirox, fluconazole, carbon source, pH, Rim101p, Ssn6p, Hog1p, Hap43p, Sfu1p, Sef1p, farnesol, core stress response

Supplementary table S3: CDR Repressed genes. Genes that were found significantly underexpressed in Gu5 by Deseq and EdgeR. Log2(ratios) are indicated, together with CGD descriptions. The CDR isolates column indicate the number of different isolates in which the gene was previously found to be underexpressed, based on the studies of Liu et al., 2007 and Znaidi et al., 2006.

ID	Name	log2(Gu5 /Gu4)	CDR isolates	orf status	description
orf19.7094	HGT12	-2,9		verified	Glucose, fructose, mannose transporter; major facilitator superfamily; role in macrophage-induced hyphal growth; detected at germ tube plasma membrane by mass spectrometry; Snf3p-induced; 12 probable transmembrane segments
orf19.5760	IHD1	-2,6	4	uncharacterised	Putative GPI-anchored protein; alkaline upregulated; greater transcription in hyphal form than yeast form; regulated by Nrg1p, Rfg1p, Tup1p; regulated by Tsa1p, Tsa1Bp in minimal media at 37; not essential for viability
NOVEL-Ca21chr2-046		-2,2			
orf19.2475	PGA26	-2,2	3	verified	GPI-anchored adhesin-like protein of the cell wall, role in cell wall integrity; required for normal virulence; transcriptionally regulated by iron; expression greater in high iron; induced during cell wall regeneration; Hap43p-repressed
orf19.6169		-2,2	1	uncharacterised	Putative fungal-specific transmembrane protein; induced by Rgt1p
orf19.3902		-2,1	3	uncharacterised	Predicted ORF in Assemblies 19, 20 and 21; decreased transcription is observed upon fluphenazine treatment or in an azole-resistant strain that overexpresses CDR1 and CDR2

orf19.4215	FET34	-2,0	2	uncharacterised	Putative multicopper ferroxidase; expression greater in low iron and reduced in a fluconazole-resistant isolate; downregulated by Sfu1p, Hog1p; alkaline upregulated by Rim101p; ciclopirox olamine, ketoconazole and hypoxia induced
orf19.270		-2,0		uncharacterised	none
NOVEL-Ca21chr4-013	TF4C3	-1,9			
orf19.2060	SOD5	-1,9	4	verified	Copper- and zinc-containing superoxide dismutase; protective role against oxidative stress; induced by neutrophil contact, hyphal growth, caspofungin, osmotic or oxidative stress; member of a gene family including SOD1, SOD4, SOD5, and SOD6
orf19.7219	FTR1	-1,7	4	verified	High-affinity iron permease; required for mouse virulence, low-iron growth; iron, amphotericin B, caspofungin, ciclopirox, Hog1p, Sef1p, Sfu1p, and Hap43p regulated; complements S. cerevisiae ftr1 iron transport; Hap43p-repressed
orf19.5025	MET3	-1,6	4	uncharacterised	ATP sulfurlyase of sulfate assimilation; repressed by Met or Cys, Sfu1p, or in fluconazole-resistant isolate; strongly induced on biofilm formation, even in presence of Met and Cys; Hog1p-, caspofungin-, white phase-induced
NOVEL-Ca21chrR-103	TFRW266	-1,5			

orf19.6249	HAK1	-1,5	2	uncharacterised	Putative potassium transporter; similar to Schwanniomyces occidentalis Hak1p; amphotericin B induced; transcriptionally induced upon phagocytosis by macrophage; Hap43p-repressed gene
orf19.1264	CFL2	-1,5	2	uncharacterised	Putative oxidoreductase, iron utilization; regulated by Sfu1p, Sef1p, Hap43p, Nrg1p, Tup1p, Rim101p; alkaline, low iron, fluphenazine, ciclopirox olamine, flucytosine, fluconazole, biofilm induced; caspofungin, amphotericin B repressed
orf19.689	PLB1	-1,4		verified	Phospholipase B; required for host cell penetration and virulence in a mouse systemic infection; Hog1p-induced; predicted signal sequence, N-glycosylation, and Tyr phosphorylation site; expression higher in fluconazole-resistant strains
orf19.3475		-1,4	4	uncharacterised	Described as a Gag-related protein; hyphal induced; downregulation correlates with clinical development of fluconazole resistance; repressed by nitric oxide, 17-beta-estradiol, ethynyl estradiol
orf19.3664	HSP31	-1,4		uncharacterised	Putative 30 kda heat shock protein; repressed during the mating process
orf19.7296		-1,3	4	verified	Putative cation conductance protein; similar to stomatin mechanoreception protein; plasma-membrane localized; induced by Rgt1p
orf19.2048		-1,3		uncharacterised	Transcription is positively regulated by Sfu1p; Hap43p-repressed gene; planktonic growth-induced

orf19.34	GIT1	-1,2	1	verified	Glycerophosphoinositol permease, involved in utilization of glycerophosphoinositol as a phosphate source; fungal-specific (no human or murine homolog); transcription negatively regulated by Rim101p; virulence-group-correlated expression
orf19.1691		-1,2		verified	Plasma-membrane-localized protein; filament induced; Hog1p-, ketoconazole-, fluconazole- and hypoxia-induced; regulated by Nrg1p, Tup1p, Upc2p; increased expression in response to prostaglandins; biofilm- and planktonic growth-induced
orf19.4749		-1,2	4	uncharacterised	Hap43p-induced gene; hyphal-induced expression, regulated by Cyr1p, Ras1p, Efg1p
orf19.2158	NAG3	-1,2	2	verified	Putative transporter of the major facilitator superfamily (MFS); similar to Nag4p; required for wild-type mouse virulence and cycloheximide resistance; in gene cluster that includes genes encoding enzymes of GlcNAc catabolism
orf19.670.2		-1,1	4	uncharacterised	Hap43p-repressed gene; hypoxia downregulated, ketoconazole induced; late-stage biofilm-induced
orf19.2462	PRN3	-1,1		uncharacterised	Protein similar to pirin; transcriptionally activated by Mnl1p under weak acid stress; Hap43p-repressed gene
orf19.7276.1	TLO4	-1,1		uncharacterised	Member of a family of telomere-proximal genes of unknown function; transcription is upregulated in an RHE model of oral candidiasis; Hap43p-repressed gene

orf19.4450.1		-1,1	3	uncharacterised	Protein conserved among the fungal CTG-clade; gene contains two adjacent upstream SRE-1 elements; highly up-regulated in cecum-grown cells in a Cph2p-dependent manner; Hap43p-repressed gene; biofilm-induced
orf19.4779		-1,1	3	uncharacterised	Putative transporter; slightly similar to the Sit1p siderophore transporter; Gcn4p-regulated; fungal-specific (no human or murine homolog); transcriptionally activated by Mnl1p under weak acid stress
orf19.3803	MNN22	-1,0		uncharacterised	Putative Golgi alpha-1,2-mannosyltransferase; regulated by Tsa1p, Tsa1Bp in minimal media at 37 deg; Hog1p-induced; induced by nitric oxide; downregulated in core stress response; planktonic growth-induced gene
orf19.7668	MAL2	-1,0		verified	Alpha-glucosidase that hydrolyzes sucrose; required for sucrose utilization; transcriptionally regulated by Suc1p; expression induced by maltose, repressed by glucose; transposon mutation affects filamentous growth; upregulated in RHE model
NOVEL-Ca21chrR-106	TFRW269	-1,0		NA	NA

orf19.5806	ALD5	-1,0	2	verified	NAD-aldehyde dehydrogenase; decreased expression in fluconazole-resistant isolate, or in hyphae; biofilm induced; fluconazole-downregulated; protein abundance is affected by URA3 expression in the CAI-4 strain; stationary phase enriched
NOVEL-Ca21chrR-100	TFRC243	-1,0		NA	NA
orf19.7331	FCY24	-1,0		uncharacterised	Putative transporter; more similar to S. cerevisiae Tpn1p, which is a vitamin B6 transporter, than to purine-cytosine permeases; transcription is regulated by Nrg1p
orf19.2356	CRZ2	-1,0	1	verified	Putative zinc finger transcription factor; similar to S. cerevisiae Crz1p; homozygous crz1, not crz2, null mutation suppresses fluconazole resistance of homozygous cka2 null (defective in CK2 kinase); Rim101p-downregulated at pH 8
orf19.1097	ALS2	-0,9		verified	ALS family protein; role in adhesion, biofilm formation, germ tube induction; expressed at infection of human buccal epithelial cells; putative GPI-anchor; induced by ketoconazole, low iron and at cell wall regeneration; regulated by Sfu1p
orf19.2020	HGT6	-0,9	2	verified	Putative high-affinity glucose transporter of major facilitator superfamily; 20 members of C. albicans glucose transporter family; 12 probable membrane-spanning segments; core stress response, fluconazole-induced; biofilm-induced

orf19.2179	SIT1	-0,9	1	verified	Transporter of ferrichrome siderophores, not ferrioxamine B; required for wild-type invasion of human epithelial cells in vitro, but not for wild-type systemic infection in mice; transcription regulated by iron, Sfu1p, SRfg1p, Tup1p, Hap43p
orf19.2079	PHHB	-0,9		verified	Transposon mutation affects filamentous growth; late-stage biofilm-induced gene
orf19.5673	OPT7	-0,8	4	verified	Putative oligopeptide transporter; possibly transports GSH or related compounds; induced by biofilm formation; Hog1p-induced; expression of OPT6, -7, or -8 does not suppress defect of mutant lacking OPT1-3; fungal-specific; Hap43p-repressed
orf19.2461	PRN4	-0,8	4	uncharacterised	Protein with similarity to pirins; increased transcription is observed upon benomyl treatment
orf19.7106	VPS70	-0,8		uncharacterised	Ortholog(s) have role in protein targeting to vacuole
orf19.24	RTA2	-0,8	1	verified	Putative flippase required for sphingolipid long chain base release; mediates calcineurin-dependent resistance to azoles; stress-associated protein; Ca2+, calcineurin-regulated; ketoconazole, caspofungin induced; Plc1p-regulated
orf19.1121		-0,8	2	uncharacterised	none

orf19.7561	DEF1	-0,8	4	verified	Regulator of RNA polymerase II; required for filamentous growth and epithelial cell escape, dissemination in RHE model; induced by fluconazole, high cell density; Efg1p/hyphally regulated; role in adhesion and hyphal growth on solid surface
orf19.638	FDH1	-0,7	4	uncharacterised	Formate dehydrogenase, oxidizes formate to produce CO2; Mig1p regulated; induced by macrophages; fluconazole-downregulated; downregulated by Efg1p under yeast, not hyphal, growth conditions; predicted cytosolic; stationary phase enriched
orf19.4943	PSA2	-0,7		uncharacterised	Mannose-1-phosphate guanyltransferas; macrophage-downregulated gene; stationary phase enriched protein; Hap43p-repressed gene
orf19.6595	RTA4	-0,7		verified	Protein similar to S. cerevisiae Rsb1p, involved in fatty acid transport; transposon mutation affects filamentous growth; alkaline downregulated; caspofungin induced; possibly an essential gene; Hap43p-repressed
orf19.4456	GAP4	-0,7	2	verified	Putative amino acid permease; hyphal induced; regulated by Hap43p, Gcn2p and Gcn4p; shows colony morphology-related gene regulation by Ssn6p; detected at plasma membrane of yeast-form and germ tube by mass spectrometry

orf19.3406		-0,7	2	uncharacterised	Predicted ORF in Assemblies 19, 20 and 21; member of conserved Mcm1p regulon
orf19.993		-0,7	3	uncharacterised	none
orf19.4664	NAT4	-0,7		verified	Putative histone acetyltransferase, involved in regulation of white-opaque switching; early-stage biofilm-induced gene
orf19.7502		-0,7		uncharacterised	Hap43p-induced gene; greater mRNA abundance observed in a cyr1 homozygous null mutant than in wild type
orf19.411		-0,7		uncharacterised	Protein similar to GTPase regulators; expression greater in low iron; transcriptionally activated by Mnl1p under weak acid stress; Hap43p-, Sfu1p- and Sef1p-regulated; biofilm- and planktonic growth-induced
orf19.5572		-0,7		uncharacterised	none
orf19.7027		-0,7		uncharacterised	none
orf19.5455		-0,6		uncharacterised	Ortholog(s) have mRNA binding activity and role in microautophagy, polyphosphate metabolic process, vacuolar transport, vacuole fusion, non-autophagic
orf19.22		-0,6		uncharacterised	Protein with homology to peroxisomal membrane proteins; Sef1p-, Sfu1p-, and Hap43p-regulated gene
orf19.7437		-0,6		uncharacterised	Putative protein of unknown function; Hap43p-repressed gene; ortholog of *S. cerevisiae* YJL218W
orf19.4784	CRP1	-0,6		verified	Plasma membrane copper transporter; CPx P1-type ATPase; mediates Cu resistance; similar to proteins of Menkes and Wilson disease; copper-induced; Tbf1p-activated; suppresses Cu sensitivity of S. cerevisiae cup1 mutant; biofilm-induced

orf19.2947	SNZ1	-0,6	4	verified	Stationary phase protein; soluble in hyphae; induced on yeast to hyphal switch, in response to 3-aminotriazole, or in azole-resistant strain overexpressing MDR1; regulated by Gcn4p, macrophage; no human/murine homolog
orf19.6637		-0,6		uncharacterised	Biofilm- and planktonic growth-induced gene; induced by hypoxia
orf19.333	FCY2	-0,6	1	verified	Purine-cytosine permease of pyrimidine salvage; mutation associated with resistance to flucytosine in clinical isolates; transposon mutation affects filamentation; farnesol-upregulated in biofilm; planktonic growth-induced
NOVEL-Ca21chr4-040	TF4W15	-0,6			
orf19.6276		-0,6	1	uncharacterised	none
orf19.5079	CDR4	-0,6		verified	Putative transporter of ATP-binding cassette (ABC) superfamily; biofilm, fluconazole, Sfu1p, Hog1p, core stress response induced; caspofungin repressed; fluconazole resistance is not affected by mutation or correlated with expression
orf19.4914.1		-0,6		uncharacterised	none
NOVEL-Ca21chrR-026	TFRW21	-0,6			
orf19.1510	SDF1	-0,6	3	uncharacterised	Protein not essential for viability
orf19.822		-0,6		verified	Similar to heat-shock protease protein; protein detected in some, not all, biofilm extracts; fluconazole-downregulated; transcription induced in cyr1 or ras1 homozygous null mutant; stationary phase enriched protein; Hap43p-repressed gene

orf19.6474		-0,6		verified	Membrane-localized protein
orf19.6078	POL93	-0,6		uncharacterised	Predicted ORF in retrotransposon Tca8 with similarity to the Pol region of retrotransposons encoding reverse transcriptase, protease and integrase; downregulated in response to ciclopirox olamine; induced upon biofilm formation
orf19.768	SYG1	-0,6	1	uncharacterised	Ortholog(s) have role in signal transduction and mitochondrion, plasma membrane localization
orf19.1971		-0,6	1	uncharacterised	Has domain(s) with predicted zinc ion binding activity
orf19.2803	HEM13	-0,6		verified	Coproporphyrinogen III oxidase; antigenic; localizes to yeast cell surface, not hyphae; iron-regulated expression; macrophage-downregulated; not Rfg1p regulated, farnesol-induced; possibly essential; Hap43p-repressed gene; biofilm-induced
orf19.1868	RNR22	-0,5		uncharacterised	Putative ribonucleoside diphosphate reductase; shows colony morphology-related gene regulation by Ssn6p; RNA abundance regulated by tyrosol and cell density; Hap43p-repressed gene; biofilm- and planktonic growth-induced
orf19.434	PRD1	-0,5		uncharacterised	Putative proteinase; transcription is regulated by Nrg1p, Mig1p, and Tup1p; Hog1p-induced; stationary phase enriched protein; Hap43p-repressed gene

orf19.3441	FRP6	-0,5		uncharacterised	Putative ammonia transport protein; transcription is regulated by Nrg1p and Tup1p; regulated by Ssn6p; upregulated in the presence of human neutrophils; planktonic growth-induced gene
orf19.4780	MFS	-0,5		uncharacterised	Predicted membrane transporter, member of the drug:proton antiporter (12 spanner) (DHA1) family, major facilitator superfamily (MFS)
orf19.5680		-0,5	2	uncharacterised	Predicted ORF in Assemblies 19, 20 and 21; possibly an essential gene, disruptants not obtained by UAU1 method
orf19.3419	MAE1	-0,5		uncharacterised	Malic enzyme, mitochondrial; transcription regulated by Mig1p and Tup1p; shows colony morphology-related gene regulation by Ssn6p; Hap43p-repressed gene
orf19.2069	SMF3	-0,5		verified	Putative vacuolar iron transporter; alkaline upregulated; caspofungin repressed; transcriptionally activated by Mnl1p under weak acid stress; Hap43p-repressed gene
orf19.6079		-0,5		uncharacterised	Predicted ORF in retrotransposon Tca8 with similarity to the Gag region encoding nucleocapsid-like protein; transcription is downregulated in response to ciclopirox olamine; filament induced; regulated by Rfg1p, Tup1p; overlaps orf19.6078.1
orf19.386	SAM4	-0,5	2	uncharacterised	Putative S-adenosylmethionine-homocysteine methyltransferase; Hap43p-repressed gene; alkaline upregulated

orf19.7459		-0,5		verified	Putative mitochondrial protein with a predicted role in respiratory growth; mutants display a strong defect in biofilm formation; fluconazole-induced; ketoconazole-repressed
orf19.7551	ALO1	-0,5	1	verified	D-Arabinono-1,4-lactone oxidase involved in biosynthesis of dehydro-D-arabinono-1,4-lactone, which has a protective role against oxidative damage; plasma membrane-localized;required for full virulence in a mouse model of systemic infection
orf19.3915		-0,5	2	uncharacterised	Putative metallodipeptidase; protein present in exponential and stationary growth phase yeast cultures; Hog1p-induced; Hap43p-repressed; sumoylation target
orf19.125	EBP1	-0,5		verified	NADPH oxidoreductase; interacts with phenolic substrates, such as 17beta-estradiol; possible role in response to estrogen; induced by oxidative and weak acid stress, nitric oxide, benomyl; activated by Cap1p, Mnl1p; Sko1p-, Hap43p-repressed
orf19.7323	CBP1	-0,4		verified	Corticosteroid binding protein; transcription induced at late log-phase or upon adherence to polystyrene; not induced by corticosterone; contains a possible NAD/FAD binding region; regulated by Nrg1p, Tup1p

orf19.2396	IFR2	-0,4	2	uncharacterised	Zinc-binding dehydrogenase; upregulated by benomyl, ciclopirox olamine or alpha pheromone; regulated by oxidative stress (via Cap1p) and osmotic stress (via Hog1p); protein present in exponential and stationary phase; Hap43p-induced gene
TF3W232		-0,4			

Supplementary table S4: **List of the strains used in the study**

Strains	Descriptions	Reference
CAI4	ura3/ura3 :: imm434	Fonzi, W. A., et al. 1993
Gu4	Fluconazole susceptible isolate	Franz, R., M., et al., 1999
Gu5	Fluconazole resistant isolate overexpressing CDR1/CDR2	Franz, R., M., et al., 1999
F2	Clinical isolate from patient F, Fluconazole sensitive	Franz et al., 1998
F5	Clinical isolate from patient F, Fluconazole resistant, MDR1 overexpressing	Franz et al., 1998
G2	Clinical isolate from patient G, Fluconazole sensitive	Franz et al., 1998
G5	Clinical isolate from patient G, Fluconazole resistant, MDR1 overexpressing	Franz et al., 1998
DSY290	Azole sensitive clinical isolate	Singh, A., et al., 2011
DSY292	azole resistant clinical isolate due to overexpression of CDR1	Singh, A., et al., 2011
DSY544	Azole sensitive clinical isolate	Singh, A., et al., 2011
DSY775	azole resistant clinical isolate due to overexpression of CDR1	Singh, A., et al., 2011
DSY347	Azole sensitive clinical isolate	Singh, A., et al., 2011
DSY289	azole resistant clinical isolate due to overexpression of CDR1	Singh, A., et al., 2011
CAF12	*ura3D::imm434/ura3D::imm434 (URA3)*	W. A. Fonzi, et al 1993
CAYC2	*ura3D::imm434/ura3D::imm434, als1D::hisG/als1D::hisG-URA3-hisG*	Fu, Y., et al., 2002
CDH15	*CAI-4 but mnn4□::hisG/mnn4□::hisG, RPS10::URA3*	Hobson, R.P., et al., 2004
CKY101	*CAI-4, ade2 ::pDBI52*	Brown, D.H., et al., 1999

CKY230	*CAI-4, Dcz f1 ::hisG /Dcz f1 ::hisG, ade2 ::pDBI52*	Brown, D.H., et al., 1999
CKY116	*ura3::imm434/ura3::imm434 CZF1/czf1::hisG -URA3-hisG*	Langford, M.L., et al., 2010
TW1	Clinical oral isolate	White, T.C., et al., 1997
TW8	Clinical oral isolate with higher expression of ERG16, CDR1, MDR1	White, T.C., et al., 1997
TW9	Clinical oral isolate with higher expression of ERG16, CDR1, MDR1	White, T.C., et al., 1997
TW17	Clinical oral isolate with higher expression of ERG16, CDR1, MDR1	White, T.C., et al., 1997

Supplementary table S5: **Sequences of the primers used in the study**

Gene name	Primer sequence
MNN4	FOR: AGTAGCTCATGGAGGTCCAC<br>REV: TCCCAGGGGAATGCCATACCAT
WH11	FOR: CGAATCCAAATTAACTCCAGATTCTC<br>REV: CAGAAGTAGCTTTACCAGCAGCACT
ORF19.6713	FOR: ACCACTTCGACAACATCCTC<br>REV: GCAGTTCCAGCGGGCGTAA
YOR1	FOR: ACACCCCCACCTAATTCAGATGCT<br>REV: CAAATCATCGGGTTGCAAAGTTCGT
ALS1	FOR: CGCTCCACCTGGTGAAACCGA<br>REV: TGGTGGTGCAGTAACAGTGGTGG
HAP41	FOR: TGTTTTCCCACCCATCAATACCACC<br>REV: TGATGCGCACATGGTGGGGG
SCH9	FOR: GCACCACCGAATATTTAGCCCCCG<br>REV: TCTCTAGCATCATCTGTGGCCCCT
CZF1	FOR: GAGCAGTGCCAACGTCAGGGTC

	REV: GCTTCTCTGTTGCGCCGTT
ORF19.3769	FOR: AAAGCGTCTTCTGCCTTCACCGG<br>REV: TAGCCGATGATGTGTCATTGGGGAC
CDR4	FOR: GGGCTTCCGGTGCGGG<br>REV: GGTTGCCGCAAGTACG
RTA4	FOR: CCATTGCTGCCACTTATGTTCCAG<br>REV: GGTGGCGGTGGCGGTGTTAA
EBP1	FOR: CCTCTTATTGCGCCATCAGC<br>REV: GCTTCAAGTGCATGTTTAG
CBP1	FOR: GTGTTCATAACGGGAAAGCA<br>REV: GGGTTCGTTGTCCAATCGGTGAC
ORF19.4780	FOR: AGCCCTTGTCCCACTCCCATCT<br>REV: CCATTGGGCACCACGTTGGGT
CDR1	FOR: AAGAGAACCATTACCAGG<br>REV: AGGAATCGACGGATCAC
NGuT-chr3-105	FOR: CCCCAACCAGGTAATCACCTCGAG<br>REV: AGGTAAAATGGCCCCCGACAA
NGuT-chrR-157	FOR: ATCAAACGTGAAATCATGGGCGGA<br>REV: TCCGTCCTCTTAGACCTTGTTTGCC
NGuT-chr3-128	FOR: ACTTCTGGGTATACCGGTTGGCA<br>REV: ACAAAGGCCTTAGGTGGGGACT
NGuT-chr4-123	FOR: ACCACTTCGACAACATCCTC<br>REV: GCAGTTCCAGCGGGCGTAA
TF3W232	FOR: GATCAAACTCTGTCCGATTAC<br>REV: CTTTACCAAAACACTAG
TF4W15	FOR: GCAGCAGCTCCCAAGCG<br>REV: CCAGCCGCATTACAAATACG

TFRW269	FOR: GCAATGTGCTGTCCATGG<br>REV: GCCTCTAATGATGTGTGAATAC
MDR1	FOR: GGCGGATTTACTCCTGATACAACTC<br>REV: GCGACGGGCTGTTGAGTAAACTAT
UPC2	FOR: TCCATCCTTGACCCCTAGTCCT<br>REV: CGGCTGAGTTTTGATGTCTTGA
PDR16	FOR: CGAGCTACTAAATGGCATGAATCTG<br>REV: CAAATGGTTCGGATATACCAAATTCA
HSP104	FOR: GCCGCCACCGTGCAAGAAAC<br>REV: CCACAGCAACAGCAGCAGCAC
CMP1	FOR: ACTTGGTCATTACCGTTTGTGGGTG<br>REV: CCTTCTCTTCTTCGTTGGCTCT
CNB1	FOR: ATGGGGGCTAACGCAAGTATTCTTG<br>REV: TCAGAACATATTTAATGTCAAAGTG
CRZ1	FOR: CAGGATGATGGGTCACAGC<br>REV: ACAGTAGGTGCCGGTGGAGGT
CKA2	FOR: AGTAGGGTGTATGTTGGGTGCCA<br>REV: TCCATTGCCTCTTTAGCTGTTGGTC
HSP90	FOR: ACTTGTTGATGCTCCAGCTGCCA<br>REV: CCAGCTGGTTCGTCAGTTGAGGC
HOG1	FOR: TGGGCTCACCTCCTGCTGATGT<br>REV: TCCACTGGCAAGTCTGCGTCA
GSC1	FOR: TGCTTCGTCAAGATGGGCTGCT<br>REV: CACCCAATGGCATGACGGC
GSL1	FOR: TGGGGGAGGTAGCGTCACCG<br>REV: ACCATTTGCCAGACCACCATGCC
GSL2	FOR: CCGGCGTTAGACAACCAACCCA<br>REV: GTCACAAACAGACGCTGGGGC

ACT1	FOR: GGGTAGGGTGGGAAAACTTCA<br>REV: TTGAAACCACTGCCGACAGA
ADAEC 5’UTR	FOR: GTGCACCCCCTCACACACAAACAC<br>REV: GGGGAGGAGGTGGAGGAGGAGAA
ADAEC ORF	FOR: TGCCATCATCAGCTGCTCCTGC<br>REV: CGGTGGTGTCTTCATCTGCGCC
TAC1 5’UTR	FOR: CAGCAGCAACAACAGCAGGAGTACC<br>REV: GGAGGTGGTGGTGGTGGTGAAAG
TAC1 ORF	FOR: GCACGTCAATTAGGCGAGACAC<br>REV: TGCTGGTGAACGACCTGTGCT
